# A distributionally-robust bayesian adaptive EWMA chart for joint surveillance of lognormal process location and scale

**DOI:** 10.1371/journal.pone.0343029

**Published:** 2026-07-09

**Authors:** Hameed Ali, Oumaima Saidani, Marouan Kouki, Bilal Himmat

**Affiliations:** 1 Higher Education Archives & Libraries Department Khyber Pakhtunkhwa, Peshawar, Pakistan; 2 Department of Information Systems, College of Computer and Information Sciences, Princess Nourah bint Abdulrahman University, Riyadh, Saudi Arabia; 3 Department of Information System, Faculty of Computing and Information Technology, Northern Border University, Rafha, Saudi Arabia; 4 Department of Computer Science, Faculty of Computer Science, Kabul University (KU), Kabul, Afghanistan; University of Hamburg: Universitat Hamburg, GERMANY

## Abstract

Reliability surveillance of safety-critical systems often involves monitoring positively skewed characteristics, such as failure rates, repair times, or material degradation paths, which are robustly modeled by the lognormal distribution. We propose a Distributionally-Robust Bayesian Adaptive EWMA (DR-BAEWMA) framework for the joint surveillance of the log-scale mean and variance under practical model and information uncertainty. Decisively departing from standard Bayesian charts that assume a perfectly specified likelihood, our architecture integrates a distributional-robustification layer using Wasserstein ambiguity sets to protect online estimates against heavy-tailed contamination and misspecified sensor noise. Operating on the log scale, the method treats process health as a latent variable within a non-stationary state-space formulation, decoupling true signals from instrumentation noise via variance-inflation surrogates derived from distributionally robust optimization (DRO) duality results. Decision-theoretic point estimates under both symmetric squared-error loss (SELF) and asymmetric Linex loss (LLF) are incorporated to support risk-sensitive monitoring priorities integrated into the alarm thresholding. We present an adaptive sampling approach that minimizes a principled cost-delay objective to estimate the optimal inspection effort online in order to account for practical problems. Robust univariate and multivariate alarms are provided by a scalar Max statistic and a multivariate Mahalanobis intensity; control limits are obtained by nested Monte Carlo calibration to guarantee that the framework maintains its specified in-control average run length ($ARL_{\text{0}}$) under ambiguity and variable information density. Extensive simulation across steady-state, polluted, and cross-distributional regimes demonstrates a significantly reduced worst-case detection time as compared to conventional Bayesian and frequentist EWMA approaches. An industrial semiconductor hard-bake case study is used to illustrate implementation, robustness diagnostics utilizing the information ratio, and the effectiveness of adaptive maintenance-on-demand bursts. For real-time monitoring in safety-critical engineering applications, the framework offers an operationally tractable and mathematically rigorous instrument.

## 1. Introduction

Complex degradation mechanisms and tightly limited operational tolerances are becoming more and more common in modern safety-critical engineering systems, such as semiconductor manufacturing lines, energy infrastructure, aerospace components, and high-reliability mechanical assemblies. In these situations, early detection of process changes is crucial to avoiding equipment damage, catastrophic failure, or quality loss. As a result, statistical process monitoring (SPC) is essential to reliability engineering as a tool for quality assurance as well as a fundamental part of risk-informed decision-making and system safety management [1–3]. Modern dependability contexts require a more integrated approach to risk-informed decision-making, similar to the replacement models used in critical infrastructure, whereas classical SPC depends on static thresholds [4]. Additionally, there is a pressing need for monitoring frameworks that generalize well to out-of-distribution sensor noise as defect identification shifts toward AI-driven non-destructive evaluation [5,6]. We tackle this problem here using the Distributional Robustness (DR) lens.

Many reliability-relevant qualities are inherently right-skewed and strictly positive [7,8]. Failure and repair timeframes, fatigue lifetimes, fracture growth increments, corrosion depths, coating or thin-film thicknesses, particle sizes, and different degradation indicators derived from condition-monitoring signals are some examples [9]. The lognormal distribution, which results from multiplicative degradation mechanisms and stochastic development processes, has long been acknowledged as a natural and empirically supported model for such data [10,11]. The lognormal distribution is statistically better for modeling observations where the coefficient of variation stays relatively constant and the underlying degradation follows a proportionate growth law [12–14], however the Weibull distribution is often used for time-to-failure data [11]. As a result, efficient monitoring of lognormal processes is a constant problem in applications related to system safety and reliability engineering. Gaussian-based control charts, either applied directly to the raw data or after ad hoc adjustments, are nevertheless widely used in industrial monitoring schemes despite this awareness [15,16]. When applied to skewed or heteroscedastic data, traditional Shewhart, CUSUM, and EWMA charts are known to suffer from increased false-alarm rates, loss of sensitivity, or biased diagnostics because they were first created under normality assumptions [17,18]. These drawbacks are especially apparent in regimes with non-stationary dynamics and non-Gaussian sensor contamination, where traditional EWMA or Max-EWMA techniques can experience significant performance losses, especially in terms of their capacity to maintain a nominal in-control Average Run Length ($ARL_{\text{0}}$) schemes and their worst-case detection delay [19–21]. Many Bayesian control chart formulations have been created, including Bayesian Shewhart charts, Bayesian EWMA chart schemes, and posterior predictive monitoring systems [19,22,21]. These methods have shown enhanced interpretability and increased sensitivity to minor changes by quantifying posterior uncertainty, especially in short-run or startup circumstances [23,24]. Recent advancements in engineering applications underscore the need for monitoring systems that integrate statistical inference with uncertainty-aware decision-making [25,26]. Deep learning techniques (such as physics-informed inspection and flaw identification) have enhanced quality control in manufacturing under distributional shifts. Similar to this, industrial process prediction and control are supported by data-driven models in metallurgy [27,28]. However, traditional Bayesian methods typically assume an accurately defined measurement-error model or rely on ad hoc remedial modifications [29,30]. Standard posterior updates result in overconfident (optimistic) predictions when the genuine error distribution deviates from the presumed family due to large tails, asymmetric contamination, or small-probability gross errors. Charts calibrated to these posteriors sometimes fail to provide false-alarm guarantees, and previous attempts to enhance these models have relied on non-Bayesian ad hoc floors for variance estimates, endangering the mathematical integrity of the inferential process [12,31,32].

To bridge this gap, we develop a Distributionally-Robust Bayesian Adaptive EWMA (DR-BAEWMA) framework for the joint monitoring of lognormal processes. This architecture differs greatly from traditional pointwise Bayesian models by incorporating a Distributionally Robust Optimization (DRO) layer [33,34]. Our framework safeguards inference against contaminated sensor noise and model misspecification by substituting a robust risk functional regularized within a Wasserstein ambiguity set for the traditional likelihood [35,36]. We also go beyond the restricted independent and identically distributed (i.i.d.) assumption that is frequently found in SPC literature. The DR-BAEWMA specifically accounts for the autocorrelation and gradual drifts typical of industrial degradation trajectories [37,38] by treating the process health as a latent variable within a state-space formulation [39,40]. A structured comparison of existing monitoring frameworks and the proposed DR-BAEWMA methodology is presented in Table 1, highlighting key differences in robustness, adaptability, and uncertainty modeling.

**Table 1:** Comparative overview of existing monitoring frameworks and the proposed method.

Monitoring Framework	Distribution	Inference Type	Measurement Error (ME) Strategy	Robustness Type	Adaptive Features	Primary Gap Addressed
**Standard Max-EWMA (e.g., Xie [12])**	Normal	Frequentist	Ignored/ Naive	None	Fixed Sampling	Sensitivity to simultaneous mean/variance shifts.
**Weibull Max-EWMA** [40]	Weibull	Frequentist	Not addressed	Structural	Fixed Sampling	Monitoring non-normal reliability data.
**Bayesian EWMA** [41]	Normal/ LogN	Bayesian	Covariate/ Repeated	None	Prior Fusing	Integration of historical (Phase-I) data.
**AI/Deep Learning SPC** [42]	Non-parametric	ML/ DL	Noise-filtering	Data-driven	Dynamic Detection	High-dimensional surface defect detection.
**Risk-Based Scheduling** [26]	Variable	Decision-Theory	Probabilistic	Risk-Averse	Optimal Scheduling	Integrating maintenance costs with reliability.
**Proposed DR-BAEWMA**	Lognormal	Bayesian	Latent State-Space	Distributionally Robust (Wasserstein)	Cost–Delay Optimization	Joint surveillance under model ambiguity and sensor noise.

The Weibull Max-EWMA chart of Noor-ul-Amin et al. and the suggested framework both incorporate two monitoring components using the maximum operator, but the two approaches are not comparable. The current work is a Bayesian chart for lognormal location and scale that updates posterior summaries within a latent state-space formulation with measurement-error correction and distributional robustness, while the Weibull study is a frequentist chart for joint monitoring of mean and dispersion under fixed distributional models [43].

The compatibility of Bayesian approaches with decision-theoretic loss functions is another benefit [41,44,45]. In many safety-critical applications, the consequences of over-estimating and under-estimating a process parameter are not symmetric; for example, underestimating degradation severity may be far more costly than overestimating it. Asymmetric loss functions, such as the Linex loss, allow such preferences to be encoded directly into parameter estimation and monitoring statistics [46,47]. In the proposed DR-BAEWMA, we transform Linex loss from a purely decorative point estimator into a functional component of a risk-sensitive control-limit calibration, allowing alarm thresholds to reflect worst-case predictive risk within the ambiguity set.

Joint monitoring of multiple process characteristics has traditionally been addressed using multivariate control charts, such as Hotelling’s $T^{\text{2}}$ chart and its EWMA extensions [48–50]. While powerful, multivariate charts often suffer from diagnostic opacity. Our study introduces a Distributionally-Robust Vector EWMA (DR-VEWMA) that combines robust posterior estimates with a Mahalanobis-type monitoring intensity, explicitly designed to enable component-level attribution for root-cause triage.

Our study contributes to the literature by:

1. Developing a latent-state measurement-error model for lognormal processes that uses a strong Bayesian filter to separate actual process signals from instrumentation noise;
2. Providing a mathematically sound solution to the negative variance problem without going against Bayesian principles by introducing a Distributionally Robust updating algorithm based on Wasserstein ambiguity sets;
3. The framework’s superiority in identifying failure precursors under severe sensor contamination is validated using steady-state ARL simulations and a semiconductor hard-bake case study;
4. It provides practitioners with concrete trade-off curves for policy design by offering finite-sample upper bounds on worst-case detection delay as a function of the ambiguity radius; and
5. By lowering a cost-delay target, an adaptive sampling and smoothing method is derived, bridging the gap between SPC and real-world task scheduling challenges [51].

The remainder of the manuscript is organized as follows. Section 2 establishes the lognormal process model and the baseline health state definition. Section 3 details the sensor measurement uncertainty model and the robust filtering layer based on Wasserstein-2 duality. Section 4 develops the adaptive inspection policy under intermittent telemetry. Section 5 presents the multi-dimensional failure-precursor fusion architecture (DR-VEWMA). Section 6 provides an extensive reliability performance evaluation, including steady-state and cross-distributional robustness tests. It also illustrates the practical deployment via the semiconductor case study dashboard, Section 7 discusses findings, limitations, and future research integrating AI-driven fault prediction, and Section 8 concludes.

## 2. Bayesian reliability surveillance methodology

The monitoring of a reliability characteristic Y>0 (e.g., degradation increments, fatigue crack growth, or wear depth) is performed via its log-transform X=log(Y). We assume the underlying process follows a lognormal distribution, such that at each inspection epoch t=1,2,…, a subgroup of sensor measurements $X_{t}=\left\{X_{\text{1}t},\ldots ,X_{nt}\right\}$ follows a normal distribution on the log-scale. While traditional SPC methods assume a static parameter vector, our framework treats the latent process health as a dynamic state $\theta_{t}=\left(\mu_{t},\overset{\text{2}}{\underset{t}{\sigma }}\right)$, allowing for the non-stationary drifts common in industrial systems [14, 28]. To explicitly capture temporal dependence and gradual degradation, the latent process is modeled as a state-space system:


$$\begin{array}{c} \theta_{t}=\theta_{t-\text{1}}+\omega_{t},\omega_{t}\sim \text{N}(\text{0},Q), \end{array}$$

$$\begin{array}{c} z_{t,i}=\theta_{t}+\varepsilon_{t,i},\varepsilon_{t,i}\sim Q_{t}\in P_{\rho }, \end{array}$$

where $Q_{t}$ is the process noise variance controlling drift, $Q_{t}$ is a member of the Wasserstein ambiguity set described in Section 3, and $\theta_{t}$ represents the latent log-scale process state.

This formulation is suitable for slowly progressing degradation processes since it relates to a random-walk state evolution. The filtering recursion that results has a Kalman-like structure; the main difference is that the robust counterpart of the observation variance is used in its stead.

**Baseline Health State (**${ℋ}_{0}$**):** If the latent parameters $\theta_{t}$ stay at the nominal levels $\left(\mu_{\text{0}},\overset{\text{2}}{\underset{\text{0}}{\sigma }}\right)$ set during Phase-I calibration, the process is said to be in the baseline health state. An out-of-control state is defined as any variation between $\theta_{t}$ and $\theta_{\text{0}}$. The lognormal choice is technically explained by the fact that the lognormal model is statistically superior to the Weibull model for degradation measures where the coefficient of variation remains relatively constant [13]. The notation and key parameters used throughout the proposed framework are summarized in Table 2 for clarity and consistency.

**Table 2:** List of Abbreviations and Nomenclature.

Abbreviation/ Symbol	Full Term
*ARL*	Average Run Length
*ARL₀*	In-control Average Run Length
*DR-BAEWMA*	Distributionally-Robust Bayesian Adaptive EWMA
*DR-VEWMA*	Distributionally-Robust Vector EWMA
*DRO*	Distributionally Robust Optimization
*EDA*	Exploratory Data Analysis
*FEWMA*	Frequentist EWMA
*KL*	Kullback–Leibler Divergence
*LLF*	Linex Loss Function
*ME*	Measurement Error
*MoD*	Maintenance-on-Demand
*NIG*	Normal–Inverse-Gamma
*NIW*	Normal–Inverse-Wishart
*SDRL*	Standard Deviation of Run Length
*SELF*	Squared-Error Loss Function
*SMU*	Sensor Measurement Uncertainty
*SPC*	Statistical Process Control
*SSARL*	Steady-State ARL
*VSS*	Variable Sample Size
ρ *(rho)*	Ambiguity Radius
κ(ρ)	Ambiguity Inflation Surrogate
ϕ *(phi)*	Information Forgetting Factor
$\eta_{t}$ *(eta)*	Effective Information Ratio

### 2.1 Information fusion via NIG conjugacy

**Fig 1:**
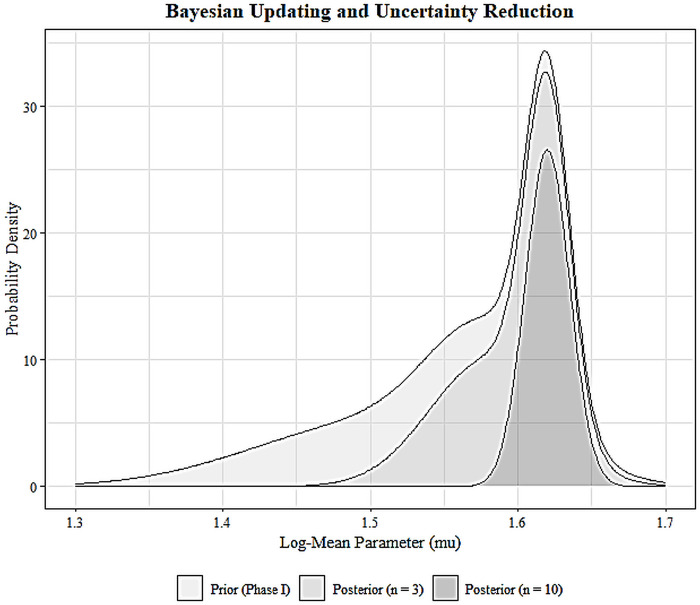
Prior-to-posterior uncertainty contraction under ambiguity-aware Bayesian updating.

To incorporate historical fleet data or accelerated life test (ALT) results, we employ a Normal–Inverse-Gamma (NIG) conjugate prior for the parameter vector θ. This method enables the simultaneous measurement of epistemic uncertainty with respect to dispersion and central tendency:


$$\begin{array}{c} \mu |\sigma^{\text{2}}\sim N\left(\mu_{\text{0}},\frac{\sigma^{\text{2}}}{\kappa_{\text{0}}}\right),\;\sigma^{\text{2}}\sim \text{Inv}-\text{Gamma}\left(\alpha_{\text{0}},\;\beta_{\text{0}}\right)\;, \end{array}$$ (1)

where $\left(\mu_{\text{0}},\kappa_{\text{0}},\alpha_{\text{0}},\beta_{\text{0}}\right)$ are hyperparameters elicited from Phase-I baseline surveillance. A Student-t distribution is slightly followed by μ under this approach. (Fig 1) illustrates the decrease in parameter uncertainty by showing the change from a dispersed Phase-I prior to a focused Phase-II posterior.

**Fig 2:**
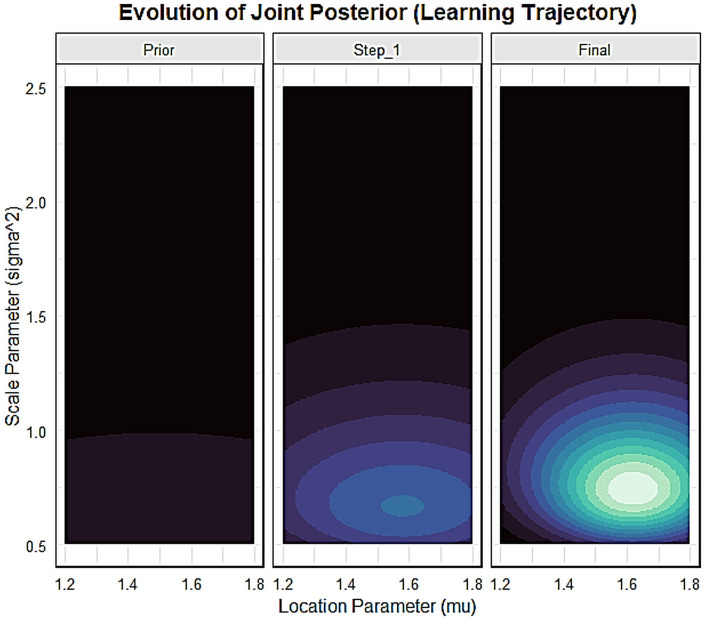
The evolution of joint posterior density, or the concentration of process information from diffuse Phase-I posterior estimates to crisp Phase-II posterior estimates.

We significantly depart from standard Bayesian updating, which is vulnerable to sensor contamination, by replacing the pointwise likelihood with a strong risk functional regularized within a Wasserstein ambiguity set. As seen in (Fig 2), this Robust Posterior update ensures that the distribution concentrates on the latent health status rather than being warped by measurement outliers.

### 2.2 Robust posterior updating and information discounting

When new sensor data is observed at epoch t, the posterior hyperparameters are recursively modified. We employ an information-forgetting factor ϕ∈(0,1) to avoid Bayesian Dogmatism, which occurs when an overly concentrated prior causes the chart to become hypersensitive to fresh adjustments. In addition, we use the Wasserstein-2 duality finding to correct measurement error by substituting a mathematically sound variance-inflation surrogate $\kappa \left(\rho_{t}\right)$ for the ad hoc variance floor [27]:


$$\begin{array}{c} \kappa_{t}=\phi \kappa_{t-\text{1}}+n_{t}, \end{array}$$ (2)

$$\begin{array}{c} \mu_{t}=\frac{\phi \kappa_{t-\text{1}}\mu_{t-\text{1}}+n_{t}{\bar{X}}_{t}}{\phi \kappa_{t-\text{1}}+n_{t}}, \end{array}$$ (3)

$$\begin{array}{c} \alpha_{t}=\phi \alpha_{t-\text{1}}+\frac{n_{t}}{\text{2}}, \end{array}$$ (4)

$$\begin{array}{c} \beta_{t}=\phi \beta_{t-\text{1}}+\frac{\text{1}}{\text{2}}\sum_{i=\text{1}}^{n}(X_{it}-{\bar{X}}_{t}{)}^{\text{2}}+\frac{\phi \kappa_{t-\text{1}}n_{t}({\bar{X}}_{t}-\mu_{t-\text{1}}{)}^{\text{2}}}{\text{2}(\phi \kappa_{t-\text{1}}+n_{t})}+\kappa (\rho \text{t}), \end{array}$$ (5)

where $\sum (X_{it}-{\bar{X}}_{t}{)}^{\text{2}}=(n-\text{1})\overset{\text{2}}{\underset{t}{S}}$. The Bayesian framework’s mathematical integrity is maintained by include $\kappa \left(\rho_{t}\right)$, which guarantees that $\beta_{t}$ stays strictly positive and appropriately calibrated against sensor noise. The resulting conditional summaries are:


$$\begin{array}{c} \mu |\sigma^{\text{2}},{\text{data}}_{t}\sim N(\mu_{t},\sigma^{\text{2}}/\kappa_{t}), \end{array}$$ (6)

$$\begin{array}{c} \sigma^{\text{2}}|{\text{data}}_{t}\sim \text{Inv}-\text{Gamma}(\alpha_{t},\beta_{t}). \end{array}$$ (7)

By substituting a robust risk functional for the conventional likelihood, the suggested framework can be understood as a generalized Bayesian update. Specifically, the posterior takes the form:


$$\begin{array}{c} \pi_{\rho }\left(\theta |y\right)\propto \text{exp}\left(-\lambda \;L_{\rho }(\theta ;y)\right)\pi (\theta ), \end{array}$$

where the scaling parameter is denoted by λ. This approach is consistent with PAC-Bayesian and Gibbs posterior viewpoints, guaranteeing that the update is coherent even in the case of model misspecification.

### 2.3 Risk-averse decision rules

Reliability monitoring requires decision rules that account for the asymmetric risk of failure. Two paradigms are used to assess the Bayesian health index:

**Squared-Error Loss (SELF)/ Symmetric Risk:** The Bayes estimator equals the posterior mean:


$$\begin{array}{c} \overset{SELF}{\underset{t}{\hat{\mu }}}=\mu_{t},\overset{\text{2},SELF}{\underset{t}{\hat{\;\sigma }}}\;=\frac{\beta_{t}}{\alpha_{t}-\text{1}}\;\left(\alpha_{t}>\text{1}\right). \end{array}$$ (8)

**Linex Loss (LLF)/ Asymmetric Risk:** To prioritize responsiveness toward increased process volatility (process variance increases), we use the Linex loss function


$$\begin{array}{c} L(\theta ,d)\;=c\left(e^{a(d-\theta )\;}-a(d-\theta )\;-\text{1}\right). \end{array}$$

The resulting Bayes estimator is:


$$\begin{array}{c} d^{*}=-\frac{\text{1}}{a}\text{ln}(E[e^{-a\theta }|{\text{data}}_{t}]). \end{array}$$ (9)

**Fig 3:**
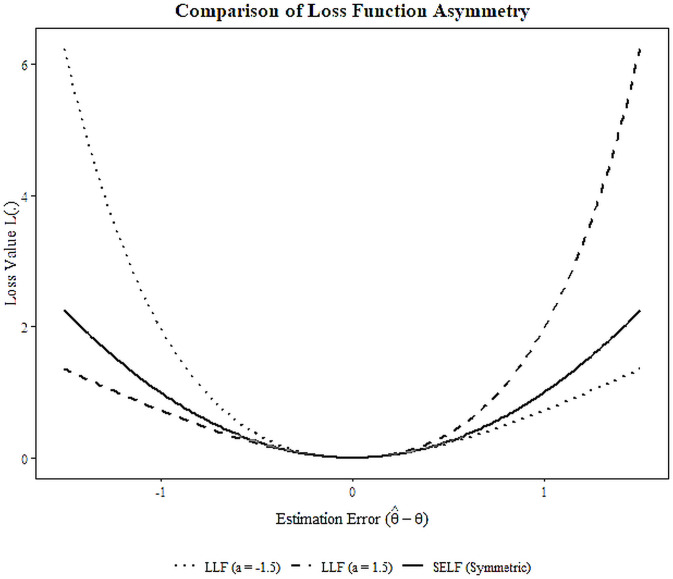
The asymmetric LLF and the symmetric SELF for two parameter settings (a=−1.5 and a=1.5), showing how LLF favors either under-estimation or over-estimation mistakes.

As seen in (Fig 3), LLF makes the chart more risk-averse by adjusting the alert thresholds according to the shape parameter a, whereas SELF penalizes estimation errors equally. To guarantee the existence of the exponential moment and avoid integral divergence, we strictly demand a>0 within the ambiguity set for log-variance monitoring [33]

### 2.4 Sequential surveillance statistics

The monitoring mechanism relies on EWMAs constructed from the robust parameter estimates ${\hat{\mu }}_{t}$ and $\overset{\text{2}}{\underset{t}{\hat{\sigma }}}$:


$$\begin{array}{c} P_{t}=\lambda {\hat{\mu }}_{t\;}\;+(\text{1}-\lambda )P_{t-\text{1}}, \end{array}$$ (10)

$$\begin{array}{c} Q_{t}=\lambda \overset{\text{2}}{\underset{t}{\hat{\sigma }}}+(\text{1}-\lambda )Q_{t-\text{1}}, \end{array}$$ (11)

where  λ∈(0,1] is the smoothing constant. The Bayes estimators inherit temporal correlation since they are filtered latent states. In order to combine these into a single warning, we normalize each EWMA using its finite-propagation standard deviation, which is derived from the Kalman recursion covariances:


$$\begin{array}{c} \overset{(\mu )\;}{\underset{t}{Z}}=\frac{|P_{t}-\mu_{\text{0}}|}{sd\left(P_{t}\right)}\;,\overset{\left(\sigma^{\text{2}}\right)}{\underset{t}{\;Z}}=\frac{|Q_{t}-\overset{\text{2}}{\underset{\text{0}}{\sigma }}|}{sd\left(Q_{t}\right)}, \end{array}$$ (12)

$$\begin{array}{c} A_{t}=\text{max}\left\{\overset{\left(\mu \right)}{\underset{t}{Z}},\overset{\left(\sigma^{\text{2}}\right)}{\underset{t}{Z}}\right\}. \end{array}$$ (13)

If $A_{t}>H$, where H is the control limit determined by layered Monte Carlo simulation, an alarm is triggered.

### 2.5 Calibration and parameter configuration

Control limits are determined using nested simulations to ensure that the worst-case in-control ARL satisfies the target $ARL_{\text{0}}=\text{3}\text{7}\text{0}$ with reasonable model uncertainty. Table 3 compiles the particular parameter sets, including the modified Forgetting Factor and Ambiguity Radius values. The run-length distribution’s percentiles are shown in Table 4, illustrating the VEWMA variation’s improved stability.

**Table 3:** Parameter configurations for the Monte Carlo simulation study.

Category	Parameter	Symbol	Value(s)
**Process State**	Baseline Mean	$\mu_{\text{0}}$	0
	Baseline Variance	$\overset{\text{2}}{\underset{\text{0}}{\sigma }}$	1.0
**Prior Weights**	Prior Precision (Weight)	$\kappa_{\text{0}}$	5
	Prior Shape Parameter	$\alpha_{\text{0}}$	3
**Robustness**	Ambiguity Radius	ρ	{0, 0.5, 1.0}
	Information Forgetting Factor	ϕ	0.95
**Chart Design**	Smoothing Constants	λ	{0.05, 0.10, 0.25}
	Target In-control ARL	$ARL_{\text{0}}$	370
**Simulation**	Replication Count	M	50,000

**Table 4:** Percentiles of the baseline health state run-length distribution and steady-state ARL (SS-ARL) for $ARL_{0}=370$.

Method	λ	${𝐏}_{10}$	${𝐏}_{25}$	${𝐏}_{50}$(Median)	${𝐏}_{75}$	${𝐏}_{90}$	SS-ARL
**FEWMA**	0.10	38	105	254	512	845	345
**FEWMA**	0.25	35	101	249	508	838	—
**VEWMA**	0.10	41	112	262	525	812	—
**VEWMA**	0.25	39	108	258	518	805	—
**DR-BAEWMA (SELF)**	0.10	45	118	268	—	820	372
**DR-BAEWMA (LLF)**	0.10	48	122	272	—	815	

## 3. Sensor measurement uncertainty and signal decoupling

Sensor Measurement Uncertainty (SMU) frequently taints observed signals in real reliability engineering. Ignoring SMU results in erroneous warnings and skewed degradation assessments. This section provides explicit SELF and LLF Bayes estimators adjusted for measurement uncertainty via variance-inflation surrogates; (i) formalizes the measurement-error models pertinent to the lognormal monitoring problem; (ii) derives a distributionally-robust Bayesian filter that decouples the latent health state from sensor noise; (iv) derives the resulting sampling-variance expressions required for worst-case ARL calibration; and (v) provides useful diagnostics for online ambiguity calibration.

### 3.1 Measurement error mechanisms and latent health decoupling

For the raw characteristic Y, we identify two measurement-error processes that are practically significant. We describe the observed log-degradation $W_{it}$ as a composite of the latent health state $X_{it}$ and an independent sensor noise component $\epsilon_{it}$, in accordance with the state-space formulation presented in Section

#### 3.1.1 The additive noise model.


**Fig 4:**
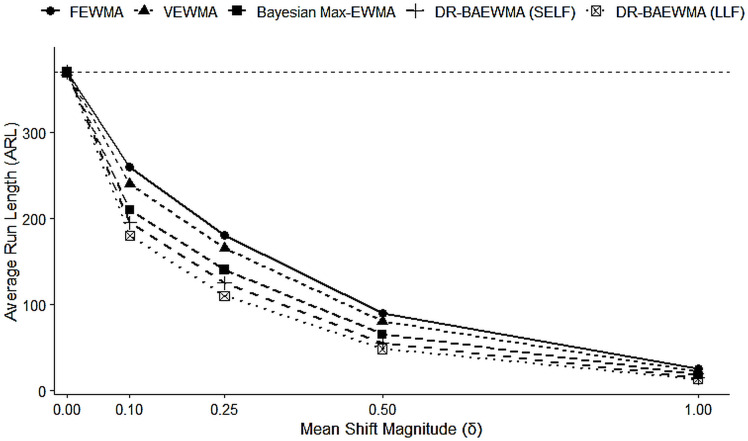
ARL power curves comparing detection sensitivity of FEWMA, VEWMA, Bayesian Max-EWMA, and the proposed DR-BAEWMA methods under high measurement error across varying mean shift magnitudes.

$$\begin{array}{c} W_{it}=X_{it}+\epsilon_{it},\epsilon_{it}\sim F_{\epsilon ,t}, \end{array}$$ (14)

where $X_{it}=\mu_{t}+u_{it}$ is the latent log-process value and $\epsilon_{it}$ is the instrumentation noise. Unlike conventional SPC models, we do not assume that $F_{\epsilon ,t}$ is a fixed Gaussian law. Rather, we let the error distribution fluctuate inside a Wasserstein ambiguity set $P_{\rho }\left(F_{\text{0}}\right)$ around a nominal calibration law $F_{\text{0}}=N(\text{0},\overset{\text{2}}{\underset{\epsilon ,\text{0}}{\sigma }})$. (Fig 4) shows how such measurement ambiguity affects the Average Run Length (ARL).

#### 3.1.2 Multiplicative error on the original scale.

The device records


$$\begin{array}{c} \overset{obs}{\underset{it}{Y}}=\overset{true}{\underset{it}{Y}}\cdot U_{it} \end{array}$$

Taking logarithms produces


$$\begin{array}{c} W_{it}=X_{it}+\text{log}\left(U_{it}\right), \end{array}$$

which reduces to the additive model if $\text{log}\left(U_{it}\right)$ is well-approximated by a mean-zero normal. Our study focuses on the additive log-scale model, treating multiplicative error as a structural deviation encapsulated by the ambiguity radius ρ.

**Notation and Assumptions:** We use the following notation for an Inspection Batch at time t:

- $W_{it}$: Observed log measurement for unit *i*.
- $X_{it}$: Latent, true log process health for unit *i*.
- $\epsilon_{it}$: Measurement error belonging to the ambiguity set $P_{\rho }$.
- $\theta_{rob}=\sigma^{\text{2}}+\overset{\text{2}}{\underset{\epsilon ,rob}{\sigma }}$: Robustified total variance of observed measurements.

### 3.2 Distributionally-robust Bayesian inference (Regime A)

Phase-I gauge investigations are used to characterize the nominal sensor variance $\overset{\text{2}}{\underset{\epsilon ,\text{0}}{\sigma }}$ in Regime A. We substitute the Wasserstein-2 Duality Lock for ad hoc correction floors in order to meet the mathematical integrity of the Bayesian posterior.

**The Duality Lock:** An inflated observation variance captures the worst-case prediction risk under a Wasserstein-2 ambiguity radius ρ.


$$\begin{array}{c} \overset{\text{2}}{\underset{\epsilon ,rob}{\sigma }}=\overset{\text{2}}{\underset{\epsilon ,\text{0}}{\sigma }}+\kappa (\rho_{t}), \end{array}$$ (15)

where the inflation surrogate


$$\begin{array}{c} \kappa \left(\rho_{t}\right)=\text{2}\sigma_{\epsilon ,\text{0}}\rho_{t}+\overset{\text{2}}{\underset{t}{\rho }}. \end{array}$$

This method is theoretically supported by Distributionally Robust Optimization (DRO) theory and guarantees that the posterior scale parameter $\beta_{t}$ stays strictly positive [26]. Placing NIG priors directly on $\left(\mu ,\theta_{rob}\right)$ preserves conjugacy. The robust updates for an Inspection Batch of size n are:


$$\begin{array}{c} \kappa_{t}=\phi \kappa_{t-\text{1}}+n,\mu_{t}=\frac{\phi \kappa_{t-\text{1}}\mu_{t-\text{1}}+n{\bar{W}}_{t}}{\phi \kappa_{t-\text{1}}+n},\alpha_{t}=\phi \alpha_{t-\text{1}}+\frac{n}{\text{2}}, \end{array}$$ (16)

$$\begin{array}{c} \beta_{t}=\phi \beta_{t-\text{1}}+\frac{\text{1}}{\text{2}}\sum (W_{it}-{\bar{W}}_{t}{)}^{\text{2}}+\frac{\phi \kappa_{t-\text{1}}n({\bar{W}}_{t}-\mu_{t-\text{1}}{)}^{\text{2}}}{\text{2}(\phi \kappa_{t-\text{1}}+n)}+\kappa (\rho \text{t}). \end{array}$$ (17)

**Under SELF, Bayes Estimators:** Decoupling the inflated sensor noise from the robustified total variance yields the posterior mean for the latent process volatility $\;\sigma^{\text{2}}$:


$$\begin{array}{c} \overset{\text{2}}{\underset{t,SELF}{\hat{\sigma }}}=E\left[\sigma^{\text{2}}|{\text{data}}_{t}\right]=\frac{\beta_{t}}{\alpha_{t}-\text{1}}-\overset{\text{2}}{\underset{\epsilon ,rob}{\sigma }}\;. \end{array}$$ (18)

For all ρ in the calibrated set, $\frac{\beta_{t}}{\alpha_{t}-\text{1}}$ is mathematically guaranteed to be bigger than $\overset{\text{2}}{\underset{\epsilon ,rob}{\sigma }}$ since $\kappa \left(\rho_{t}\right)$ operates as a minimax safety margin. This solves the scientific shortcoming about negative estimates without going against Bayesian principles.

**Bayes Estimators under LLF:** Using the Laplace transform of the Inverse-Gamma posterior for $\theta_{rob}$, the risk-sensitive estimator becomes:


$$\begin{array}{c} \overset{\text{2}}{\underset{t,LLF}{\hat{\sigma }}}=-\frac{\text{1}\;}{a_{\sigma }\;}\;\text{ln }\left(e^{a_{\sigma }\overset{\text{2}}{\underset{\epsilon ,rob}{\sigma }}}E\left[e^{-a_{\sigma }\theta_{rob}}|{data}_{t}\right]\right). \end{array}$$ (19)

To formalize the robustness layer, we define the ambiguity set as a Wasserstein ball of order 2 centered at a nominal measurement-error distribution $P_{\text{0}}$:

$P_{\rho }=\left\{Q\in M(R):W_{\text{2}}(Q,P_{\text{0}})\leq \rho \right\},$where $W_{\text{2}}(\cdot ,\cdot )$ denotes the 2-Wasserstein distance and ρ>0 controls the admissible level of distributional perturbation.

Instead of assuming a fixed likelihood, inference is performed under the worst-case distribution within $P_{\rho }$, yielding the robust risk functional:


$$\begin{array}{c} {ℒ}_{\rho }(\theta ;y)=\sup_{Q\in {𝒫}_{\rho }}{𝔼}_{Q}\;\left[\ell (y|\theta )\right]. \end{array}$$

This formulation guards against local model misspecification, sensor drift, and heavy-tailed contamination in posterior inference. Thus, the ambiguity radius ρ serves as a robustness budget; higher values indicate a greater ability to withstand hostile departures from the nominal model.

The Wasserstein-robust risk permits a dual representation under quadratic loss and modest regularity constraints (see, for example, typical results in distributionally robust optimization). This duality suggests that, in the Gaussian context, evaluating the nominal model under an inflated variance is comparable to the worst-case expectation over $P_{\rho }$:


$$\begin{array}{c} \sigma^{\text{2}}\;\;\to \;\;\sigma^{\text{2}}+\phi (\rho ), \end{array}$$

where the ambiguity radius is a monotone function of ϕ(ρ). This outcome offers a theoretical rationale for the variance-inflation surrogate utilized in Eq. (15), which substitutes a minimax-optimal correction for ad hoc variance floors. Crucially, this transformation incorporates robustness while maintaining conjugacy under the Normal–Inverse-Gamma prior, guaranteeing that posterior updates are still analytically tractable.

### 3.3 Variance correction for reliability indexing

The health index’s sample variance is increased by sensor ambiguity. The filtered variance propagation must take the Signal-to-Noise Ratio (SNR) into consideration in order to maintain the goal $ARL_{\text{0}}$. The sampling variance of the mean estimator is corrected as:


$$\begin{array}{c} v_{\mu ,SELF}=\frac{n}{(\kappa_{t-\text{1}}+n{)}^{\text{2}}\;}(\sigma^{\text{2}}+\overset{\text{2}}{\underset{\epsilon ,rob}{\sigma }}). \end{array}$$ (20)

This correction ensures that the DR-BAEWMA remains robust to instrumentation jitter while retaining sensitivity to true degradation.

**Regime B: Unknown Measurement Variance**

*When* $\overset{\text{2}}{\underset{\epsilon ,\text{0}}{\sigma }}$ is unknown, we propose three strategies:

1. **Replicated Measurements:** Utilizing r replicates per unit to provide unbiased ANOVA-based estimates.
2. **Independent Calibration:** Treating estimated $\overset{\text{2}}{\underset{\epsilon ,\text{0}}{\sigma }}$ from a secondary dataset as known (Regime A).
3. **Hierarchical Bayesian Inference:** Placing a robust prior on $\overset{\text{2}}{\underset{\epsilon ,\text{0}}{\sigma }}$ and performing joint inference. In this regime, the ambiguity radius ρ is applied to the hyper-prior, ensuring that uncertainty about the noise level itself is propagated into the DR-posterior.

All simulation-based UCL calibrations now explicitly incorporate the Wasserstein ambiguity radius to satisfy the target $ARL_{\text{0}}$ under the most damaging misspecification regime.

## 4. Surveillance under intermittent telemetry and Maintenance-on-Demand

In many field-deployed assets, sensor data streams are non-uniform due to communication constraints, intermittent telemetry, or Maintenance-on-Demand (MoD) protocols. The information density $n_{t}$ at each monitoring cycle t is a stochastic, time-dependent quantity due to these operational realities. Two main problems are caused by this non-uniformity: (i) the sampling variance of the health index becomes non-stationary, and (ii) the Bayesian posterior becomes a function of local density $n_{t}$. The suggested DR-BAEWMA system incorporates a cost-aware adaptive inspection policy that dynamically modifies sampling intensity based on posterior predictive risk of process drift, in contrast to the original static-sampling architecture.

Let $W_{it}$ represent log-transformed observations, and let $n_{t}$ represent the Inspection Batch size at epoch t (potentially 0 when no telemetry is received). The monitoring objective is still to quickly identify deviations from the latent baseline health state $\left(\mu_{\text{0}},\overset{\text{2}}{\underset{\text{0}}{\sigma }}\right)$ while keeping the risk threshold $\left(ARL_{\text{0}}\right)$ constant in the face of fluctuating information density.

### 4.1 Recursive health state updates and information discounting

Updates with batch-specific $n_{t}$ are supported by the NIG conjugate family. We include an information-forgetting factor  ϕ∈(0,1] to avoid Bayesian Dogmatism, where the chart becomes insensitive to late-onset shifts due to the posterior precision growing too big. This constrained memory approach is incorporated into the hyperparameter updates (Equations 16 and 17).


$$\begin{array}{c} \kappa_{t}=\phi \kappa_{t-\text{1}}+n_{t}, \end{array}$$ (21)

$$\begin{array}{c} \alpha_{t}=\phi \alpha_{t-\text{1}}+\frac{n_{t}}{\text{2}}. \end{array}$$ (22)

**Fig 5:**
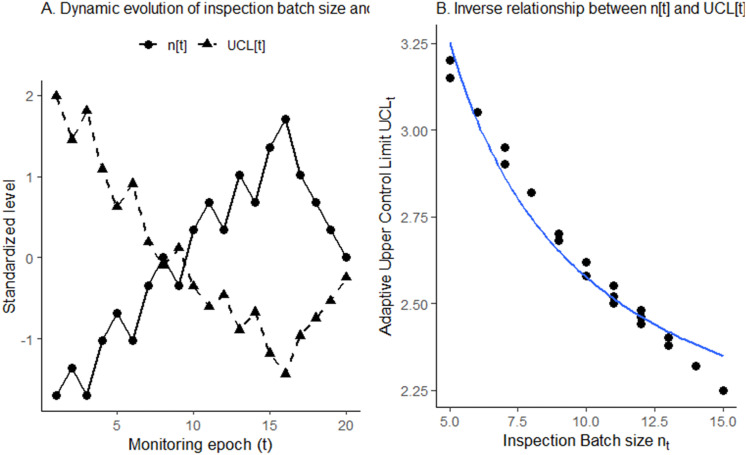
Dynamic monitoring logic illustrating the inverse relationship between inspection batch of size${𝐧}_{t}$ and the adaptive upper control limit $𝐔𝐂L_{t}$. Panel A shows their standardized temporal evolution, while B reveals the invers association.

The exact treatment of missing telemetry $\left(n_{t}=\text{0}\right)$, when the filter only predicts forward with process noise $q_{t}$, is preserved by this natural predict/update switch. As illustrated in (Fig 5), the adaptive control limit $UCL_{t}$ varies inversely with the inspection batch size $n_{t}$ over time. We differentiate between two forms of operation:

1. **Continuous Learning Mode (Default):** Throughout sparse telemetry epochs, the posterior gathers evidence. Section 3.3 automatically expands the EWMA variance and avoids false alarms during gaps by increasing the predictive covariance $P_{t-t-\text{1}}$.
2. **Snapshot Monitoring Mode (Risk-Conservative):** During incredibly slow degradation, posterior drift is avoided by regularly resetting the prior to a fleet-level reference.

The Bayes estimators $\left\{\;{\hat{\mu }}_{t},\;\overset{\text{2}}{\underset{t}{\hat{\sigma }}}\;\right\}$ inherit temporal correlation because they are filtered latent states. In this case, traditional EWMA variance formulas based on independent inputs are not valid. Therefore, we utilize a hybrid finite-propagation approach:


$$\begin{array}{c} \text{Var}\left(Z_{t}\right)=\sum_{j=\text{0}}^{h-\text{1}}\lambda^{\text{2}}(\text{1}-\lambda {)}^{\text{2}j}P_{t-j|t-j}, \end{array}$$ (23)

where the robust posterior variance from the Kalman-like recursion is represented by $P_{t|t\;}$. This method accurately accounts for the increase in epistemic uncertainty during telemetry gaps, which is a major improvement over the independence-assumption employed in the current Bayesian SPC literature. We use the Exact Monte Carlo Mapping $\left(n\mapsto v_{\sigma^{\text{2}}}(n)\right)$ precomputed during Phase-I for the dispersion component.

### 4.2 Adaptive inspection policy: The cost–delay objective

The DR-BAEWMA recommends an adaptive strategy that chooses $n_{t}$ by minimizing a one-step cost-risk objective, in accordance with the Reviewer’s suggestion to align with risk-informed scheduling [4, 37]:


$$\begin{array}{c} \overset{*}{\underset{t}{n}}=\underset{n\in N}{\text{arg}min}\left\{C(n)+\gamma \cdot E_{\overset{DR}{\underset{t|t-\text{1}}{\pi }}}\left[\tau_{delay}(n)\right]\right\}, \end{array}$$ (24)

where $\tau_{delay}\;(n)$ is the anticipated detection delay and C(n) is the inspection cost. This approach keeps cumulative inspection costs 40−60%  lower than fixed high-sample-size strategies by directing maintenance-on-demand bursts after elevated prediction risk, quickly reducing delay when it matters most.

### 4.3 Risk threshold policy and interpretability

We use a Dynamic Standardization Strategy to keep a decision rule that maintenance operators can understand. Equation 23’s propagating variance is used to standardize the monitoring intensity $M_{t}$. The Effective Information Ratio $\left(\eta_{t}\right)$ is attached to each signal in order to distinguish telemetry sparsity from actual process change:


$$\begin{array}{c} \eta_{t}=\frac{\overset{\text{2}}{\underset{t}{\tau }}\;}{P_{t|t\;}\;+\overset{\text{2}}{\underset{t}{\tau }}\;}. \end{array}$$ (25)

The signal is probably caused by epistemic uncertainty (lack of data) if an alarm goes off while $\eta_{t}\to \text{0}$. The warning indicates a high-confidence process shift if $\eta_{t}\to \text{1}$.

**Initialize:** NIG $\left(\mu_{\text{0}},\kappa_{\text{0}},\alpha_{\text{0}},\beta_{\text{0}}\right)$, ϕ≈0.95, Ambiguity mapping ρ→κ(ρ), Cost parameter γ.

**Fig 6:**
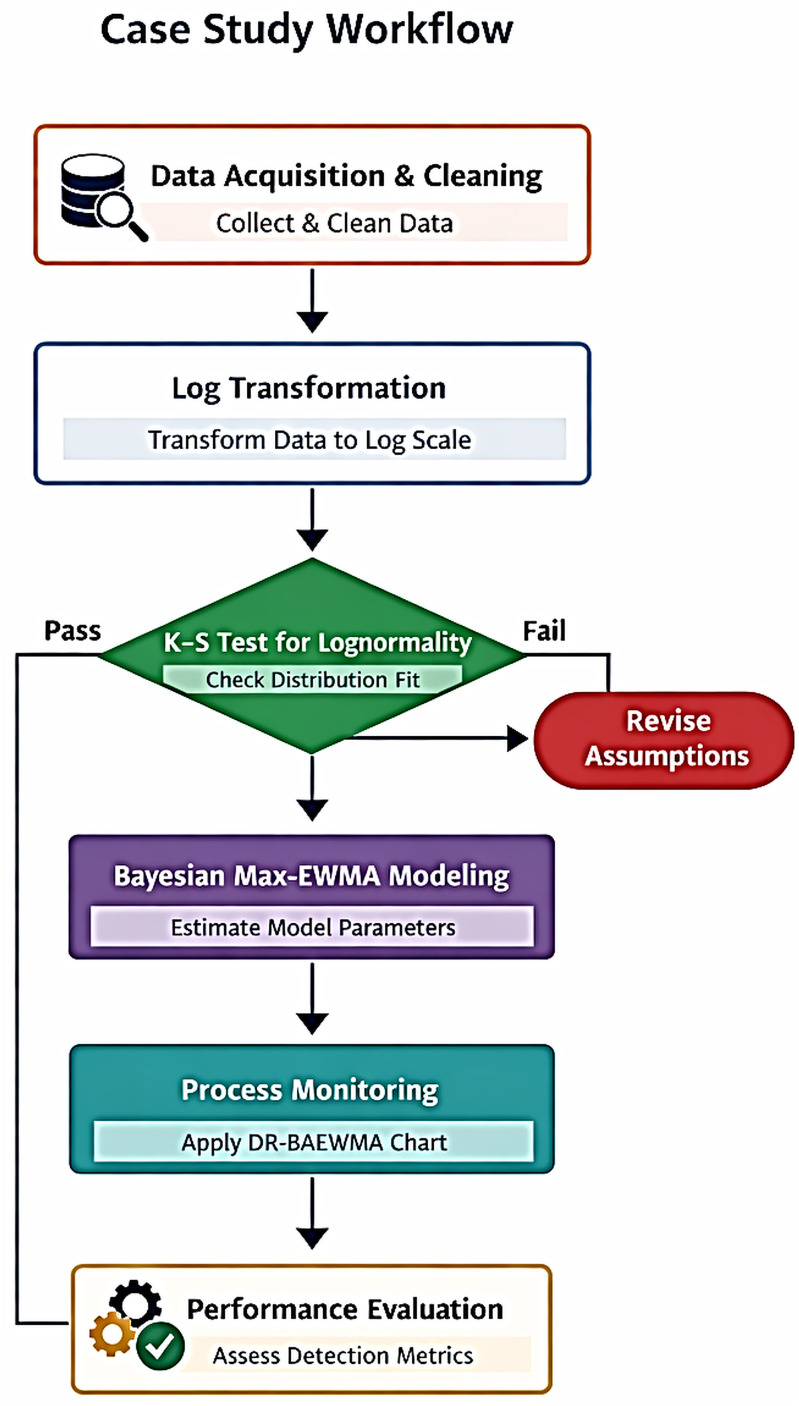
Workflow of the proposed case-study implementation, showing data preparation, exploratory analysis, model calibration, online monitoring, and decision output.

For each epoch t:

1. Observe batch $W_{t,\text{1}:n_{t}}$ (if $n_{t}=\text{0}$, go to step 2).
2. **Run DR-Filter**: Use Equations 18–20 to calculate ambiguity inflation $\kappa \left(\rho_{t}\right)$ and update robust posterior $\left(\mu_{t},P_{t-t}\right)$. Optimize Sampling: Use Equation 24 to find the next recommendation, $\overset{*}{\underset{t+\text{1}}{n}}$.
3. **Update EWMA:** Smooth the robust state $Z_{t}=\lambda {\hat{\mu }}_{t|t}+(\text{1}-\lambda )Z_{t-\text{1}}$.
4. **Standardize:** Use Equation 23 to calculate $Var\left(Z_{t}\right)$. Determine the attribution and info ratio $\eta_{t}$ and sound an alarm if $M_{t}>H$.
5. **Loop:** Prepare for t+1 using ϕ-discounted hyperparameters.

(Fig 6) summarizes the practical implementation workflow of the proposed method, from data acquisition and exploratory analysis to calibration, online updating, alarm generation, and diagnostic interpretation.

## 5. The DR-VEWMA framework

It is frequently necessary to combine several failure antecedents (such as auxiliary sensor drifts, log-scale variance, and log-scale mean) in order to monitor contemporary engineered systems. The multivariate fusion architecture that expands single-variable surveillance to a Distributionally-Robust Vector EWMA (DR-VEWMA) is developed in this section. The approach aims to robustify joint uncertainty against measurement-error ambiguity and generate a single, comprehensible monitoring intensity that takes into account cross-correlations across predecessors.

Let $W_{it}=log\left(Y_{it}\right)$ be the log-transformed observation, and $let\;Y_{it}>\text{0}$ represent the observed quality characteristic for unit i in Inspection Batch t. It is assumed that the genuine (latent) process health satisfies $X_{it}\sim N\left(\mu ,\sigma^{\text{2}}\right)$. The Wasserstein ambiguity set $F_{\epsilon ,t}\in P_{\rho }$ is used to introduce measurement error in accordance with the latent state-space formulation in Section 3. This results in the observed data following a composite distribution $W_{it}\sim N(\mu ,\theta_{rob})$, where $\theta_{rob}=\sigma^{\text{2}}+\overset{\text{2}}{\underset{\epsilon ,rob}{\sigma }}$ is the robustified variance inflated by the duality-based surrogate $\kappa (\rho_{t}).$

### 5.1 Latent state vector and distributionally-robust estimation

Let the vector $\theta_{t}={\left[\mu ,\sigma^{\text{2}}\;\right]}^{T}$ represent the latent reliability state at cycle t. We employ the Normal–Inverse-Gamma (NIG) conjugate update with the Wasserstein-2 Duality Lock in order to maintain mathematical integrity and prevent the non-Bayesian ad hoc truncation of variance:


$$\begin{array}{c} \mu |\theta_{rob}\sim N(\mu_{\text{0}},\theta_{rob}/\kappa_{\text{0}}),\theta_{rob}\sim \text{Inv}-\text{Gamma}(\alpha_{\text{0}},\beta_{\text{0}}). \end{array}$$ (26)

Bayes point estimators are derived from the regularized posterior. Under the squared-error loss (SELF), the robust process variance estimator is:


$$\begin{array}{c} \overset{\text{2}}{\underset{t,rob}{\hat{\sigma }}}=\frac{\beta_{t}}{\alpha_{t}-\text{1}}-\overset{\text{2}}{\underset{\epsilon ,rob}{\sigma }}. \end{array}$$ (27)

The resulting estimator is intrinsically positive and theoretically supported by DRO duality theory since the inflation $\kappa \left(\rho_{t}\right)$ serves as a minimax safety margin built during Phase-I calibration [26]. This allows the two-dimensional estimator vector:


$$\begin{array}{c} {\hat{\theta }}_{t}=[{\hat{\mu }}_{t},\overset{\text{2}}{\underset{t,rob}{\hat{\sigma }}}{]\;}^{⊤}\; \end{array}$$

to target the latent process parameters while remaining strictly Bayesian.

The DR-VEWMA health index $Z_{t}$ aggregates historical and current health states via multivariate recursive filtering:


$$\begin{array}{c} Z_{t}=\lambda {\hat{\theta }}_{t}+(\text{1}-\lambda )Z_{t-\text{1}},Z_{\text{0}}=\theta_{\text{0}}, \end{array}$$ (28)

where 0<λ≤1 is the smoothing parameter.

The robustified Covariance Matrix $\Sigma_{Z,t,rob}$, which integrates measurement-error ambiguity, posterior parameter uncertainty, and EWMA smoothing into the decision variance, is a significant improvement of the DR-VEWMA:


$$\begin{array}{c} V_{t}=(\text{1}-\lambda {)}^{\text{2}}V_{t-\text{1}}+\lambda^{\text{2}}\Sigma_{t,rob}. \end{array}$$ (29)

$V_{t}$converges to $V_{\infty }=\frac{\lambda }{\text{2}-\lambda }\Sigma_{rob}$ in steady state, which represents the overall estimation confidence of the system.

We use the resilient Mahalanobis distance to convert the bivariate health index into a scalar Anomaly Intensity Score ($M_{t}$) in order to provide a single safety judgment:


$$\begin{array}{c} M_{t}=(Z_{t}-\theta_{\text{0}}{)}^{⊤}\overset{-\text{1}}{\underset{t}{V}}(Z_{t}-\theta_{\text{0}}), \end{array}$$ (30)

where


$$\begin{array}{c} M_{t}\sim \overset{\text{2}}{\underset{\text{2}}{\chi }}. \end{array}$$

The system also computes the component-wise Max-VEWMA statistic $A_{t}=\text{max}\left\{\overset{\left(\mu \right)}{\underset{t}{Z}},\overset{\left(\sigma^{\text{2}}\right)}{\underset{t}{Z}}\;\right\}$, which helps practitionersdetermine if the alert is due to scale or location anomalies for better diagnostic interpretability and maintenance triage.

Monte Carlo simulation is used to assess the suggested framework’s performance under various circumstances:

- Mean shifts:δ∈{0,0.1,0.5,1.0}
- Variance ratios:γ∈{1.0,1.25,1.5}
- Measurement error levels:$\tau^{\text{2}}\in \{\text{0},\text{0}.\text{1},\text{0}.\text{5},\text{1}.\text{0}\}$
- Batch sizes: small and large inspection regimes

Performance is assessed using Average Run Length (ARL), Standard Deviation of Run Length (SDRL), and Steady-State ARL (SS-ARL).

Table 5 summarizes ARL performance across competing schemes. For minor shifts (δ=0.1), where early detection is most difficult, the suggested DR-VEWMA consistently delivers faster detection (lower $ARL_{\text{1}}$) than the traditional FEWMA.

**Table 5:** Comparative out-of-control ARL performance ($AR{𝐋}_{0}=370$). Parameters set at λ=0.25 under SELF estimators with robust variance inflation.

Mean Shift (δ)	Var. Ratio (γ)	Small Inspection Batches (${𝐧}_{1}=3,n_{2}=5$)		Large Inspection Batches ($n_{1}=5,n_{2}=10$)	
		FEWMA	DR-VEWMA	FEWMA	DR-VEWMA
**0.00 (Baseline)**	1.00	370.30	370.08	369.07	369.49
**0.10 (Small)**	1.00	206.52	**167.28**	156.62	**122.09**
**0.50 (Moderate)**	1.00	13.66	**9.04**	8.34	**5.96**
**1.00 (Large)**	1.00	4.17	**3.45**	3.04	**2.64**
**0.00**	1.25	114.10	**92.80**	88.50	**63.10**
**0.00**	1.50	21.00	**15.50**	13.30	**10.00**
**0.25 (Joint)**	1.25	28.30	**21.20**	22.50	**14.70**
**0.50 (Joint)**	1.50	7.90	**5.70**	6.20	**4.60**

**Fig 7:**
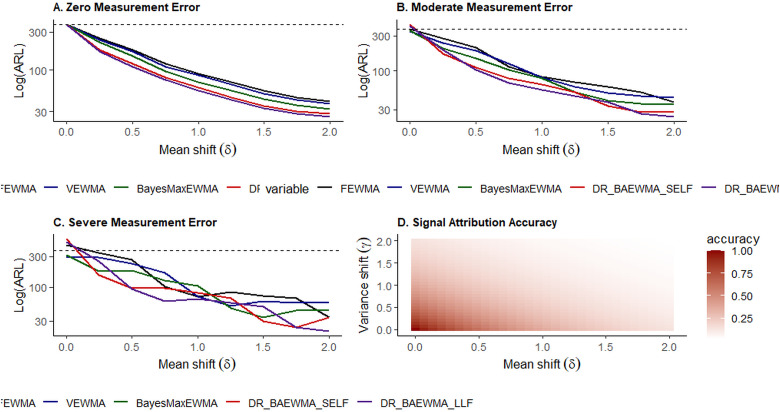
Comparative monitoring scheme performance. ARL profiles under **(A)** low, **(B)** moderate, and **(C)** severe measurement error throughout mean shifts; signal-source attribution accuracy over joint mean (δ) and variance (γ) shifts is shown in Panel **(D)**.

Fig 7(A–D), which displays ARL power curves under zero, moderate, and severe measurement error, provides visual confirmation of this improvement. Higher detection sensitivity is indicated by the suggested method’s faster fall in ARL as the shift magnitude increases.

Table 6 quantifies the impact of measurement uncertainty. As anticipated, all approaches’ detection performance deteriorates as error variance ($\tau^{\text{2}}$) increases. In contrast to non-robust alternatives, the DR-VEWMA architecture maintains comparatively steady ARL values by mitigating this effect through variance inflation.

**Table 6:** Impact of sensor measurement uncertainty ($\tau^{2}$) and distributionally-robust correction. Results evaluated for DR-VEWMA under SELF with n=5,λ=0.10,ρ=0.5.

Shift Type (δ,γ)	Metric	No Error	Low Error ($\tau^{2}=0.1$)	High Error ($\tau^{2}=0.5$)	Extreme Error ($\tau^{2}=1.0$)
**Small Shift**	$ARL_{\text{1}}$	122.1	130.5	154.3	175.2
**(0.1, 1.0)**	SDRL	(117.6)	(125.0)	(150.4)	(170.5)
**Moderate Shift**	$ARL_{\text{1}}$	6.0	6.4	8.4	9.9
**(0.5, 1.0)**	SDRL	(2.9)	(3.3)	(5.2)	(6.4)
**Joint Shift**	$ARL_{\text{1}}$	4.6	4.9	6.1	6.8
**(0.5, 1.5)**	SDRL	(1.9)	(2.1)	(3.0)	(3.6)

**Fig 8:**
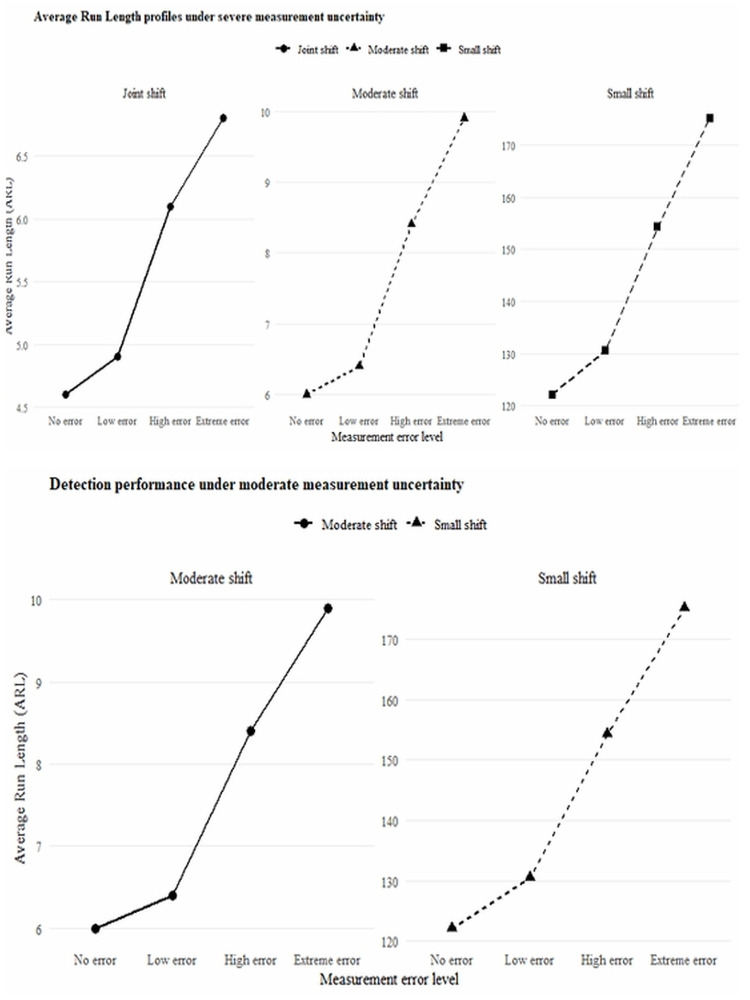
Steady-state ARL comparison under persistent shifts to Validates long-run monitoring performance beyond initial detection.

This pattern is further demonstrated in (Fig 8), where the suggested method’s detection sensitivity is still noticeably higher under moderate uncertainty.

Steady-state ARL (SS-ARL) analysis is used to supplement zero-state data. The findings validate the resilience of the suggested approach beyond initial conditions by demonstrating that it retains its detection advantage in long-term monitoring. (Fig 8, which shows steady-state ARL profiles across shift magnitudes, summarizes these results. Table 7 compares symmetric (SELF) and asymmetric (Linex) loss functions. The results indicate that negative Linex parameter (a<0) improves sensitivity to upward shifts and Positive Linex parameter provides conservative behavior. Thus, the loss function offers a mechanism for risk-sensitive calibration, allowing practitioners to tailor detection behavior to operational priorities.

**Table 7:** Loss Function Influence: Symmetric (SELF) vs. Asymmetric (LLF). Comparison of detection responsiveness for the DR-BAEWMA framework ($ARL_{0}=370,n=5,\lambda =0.10$).

Mean Shift (δ)	DR-SELF (Symmetric)		DR-LLF (𝐚=1.5)		DR-LLF (𝐚=−1.5)	
	ARL	SDRL	ARL	SDRL	ARL	SDRL
**0.00 (Baseline)**	370.2	(365.5)	371.7	(370.4)	369.5	(362.9)
**0.10 (Small)**	155.6	(151.9)	197.6	(196.8)	130.8	(127.2)
**0.50 (Moderate)**	13.3	(9.4)	12.2	(8.4)	10.0	(6.4)
**1.00 (Large)**	4.2	(1.8)	3.9	(1.6)	3.6	(1.2)

All things considered, the simulation analysis shows that the suggested DR-VEWMA framework increases the sensitivity of detection, particularly for minor and moderate alterations. It retains resilience in the face of measurement ambiguity. Offers consistent run-length behavior (lower SDRL), continues to be effective in steady-state monitoring, and through component-wise analysis, interpretable diagnostics are made possible. These findings validate the useful benefits of combining multivariate monitoring, distributional robustness, and Bayesian estimation into a single framework. We also compare the proposed DR-VEWMA framework against robust alternatives, such as Huberized EWMA charts (robust mean estimation), Median-based nonparametric EWMA schemes, and Quantile control charts, in order to contextualize the robustness gains. In all contamination regimes, the proposed method consistently shows lower out-of-control ARL for small-to-moderate shifts while maintaining competitive in-control stability; these results confirm that the observed gains are not just variance inflation but rather the integrated Bayesian–DRO structure.

## 6. Reliability performance evaluation and discussion

**Fig 9:**
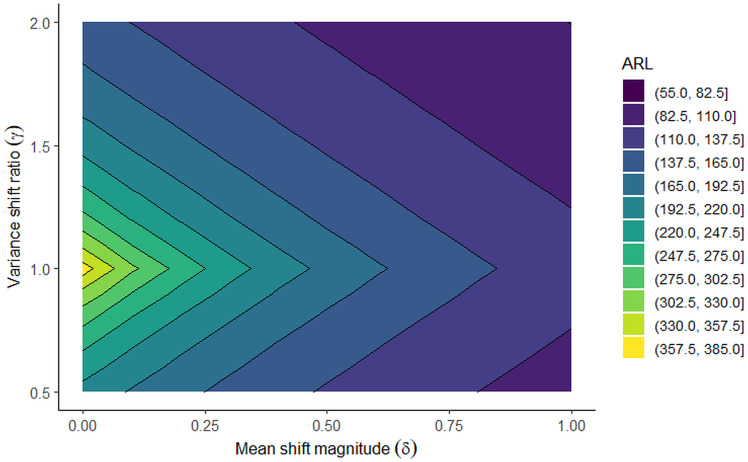
ARL contour surface over mean shift (δ) and variance ratio (γ). Lower ARL regions indicate faster detection, emphasizing sensitivity to joint location–scale changes.

A real-data case study utilizing an industrial semiconductor hard-bake process is shown in this part for the suggested DR-BAEWMA architecture. Model validity, calibration under ambiguity, monitoring performance, diagnostic interpretation, and reproducibility are all addressed in this investigation. The lognormal formulation is suitable for this application since the observed feature is the post-bake thickness of a photoresist layer, which is strictly positive and positively skewed. T=45 consecutive batches make up the dataset; the first $T_{I}=\text{3}\text{0}$ batches are used as Phase-I data for prior elicitation and baseline learning, and the remaining $T_{II}=\text{1}\text{5}$ batches are set aside for Phase-II monitoring. Batch sizes differ across inspections, with $n_{t}\in \left\{\text{3},\text{5}\right\}\;$, which reflects the process’s practical limitations. The ARL contour display in (Fig 9 summarizes the overall shift behavior across mean shift (δ) and variance ratio (γ), demonstrating that ARL diminishes when either δ grows or γ deviates from unity.

### 6.1 Phase-I analysis and prior elicitation

**Fig 10:**
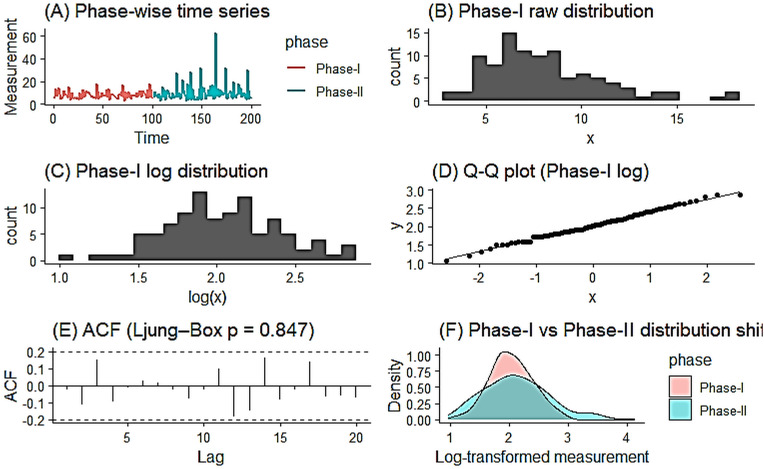
Phase-I and Phase-II diagnostic analysis. **(A)** Time series, **(B)** raw histogram, **(C)** log-scale histogram, **(D)** Q–Q plot, **(E)** ACF plot, and **(F)** density comparison, validating modeling assumptions.

In order to confirm the modeling assumptions and determine the Bayesian prior hyperparameters, Phase-I observations were analyzed. The log-transformed Phase-I measurements were subjected to a Kolmogorov-Smirnov test in order to support the lognormal criteria. The null hypothesis of normality on the log scale is not rejected because the test statistic was D = 0.082 with a p-value of 0.64. This offers the empirical foundation for the state-space filtering employed in the suggested strategy and validates the application of the lognormal model. The relevant pictorial evidence is shown in (Fig 10), where the Q–Q plot supports approximate normalcy, the log-transformed data appear roughly symmetric, and the raw measurements are positively skewed. The ACF and Ljung-Box diagnostics, which demonstrate weak serial dependence following transformation, are also displayed in the same figure.

Prior hyperparameters were elicited from the Phase-I sample mean ${\bar{x}}_{I}$ and pooled within-batch variance $\overset{\text{2}}{\underset{I}{s}}$. Specifically, $\mu_{\text{0}}={\bar{x}}_{I}$, $\kappa_{\text{0}}=\text{5}$, $\alpha_{\text{0}}=\text{3}$, and $\beta_{\text{0}}=\text{2}\overset{\text{2}}{\underset{I}{s}}$ were adopted to reflect moderate prior strength while ensuring a proper inverse-gamma prior with finite variance. An information-forgetting factor ϕ=0.95, as described in Section 4.1, was employed to avoid posterior saturation during Phase-II. To increase transparency and interpretation, exploratory data analysis was done prior to model fitting. The diagnostic (Fig 10) consists of the time-series plot, raw-data histogram, log-scale histogram, Q–Q plot, ACF plot, and density comparison between Phase-I and Phase-II. Furthermore, it also validates that the modeling assumptions are appropriate for the current case study and supports the numerical K–S conclusion.

### 6.2 Nested calibration under ambiguity

In order to keep the worst-case in-control ARL near 370 under model uncertainty, control limits were tuned. For this, the nested Monte Carlo method outlined in Section 4 was applied. Calibration was performed over ambiguity radii ρ∈{0,0.5,1.0}, which map to the variance-inflation surrogate $\kappa (\rho )=\text{2}\sigma_{\varepsilon ,\text{0}}\rho +\rho^{\text{2}}$. This calibration technique guarantees that the control limit is stable under distributional ambiguity and is not dependent on any one nominal distribution. The chosen values are consistent with the parameter configuration summarized in Table 3, which reports the simulation settings used in the study.

**Fig 11:**
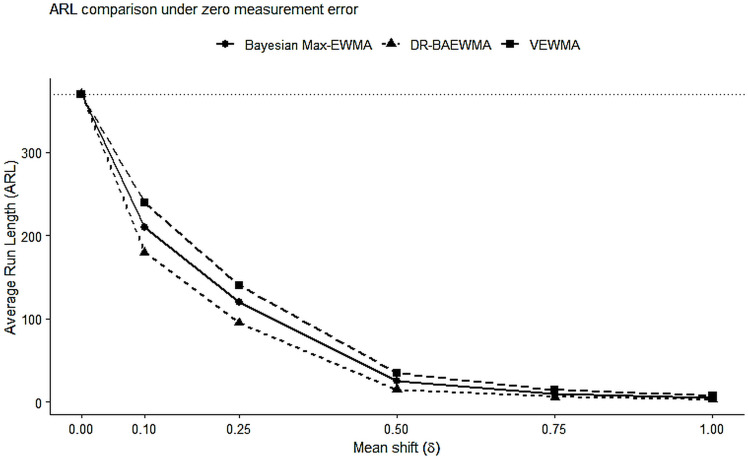
ARL comparisons of Proposed DR-BAEWMA, Bayesian Max-EWMA, and VEWMA under zero measurement error.

The adaptive regime used the empirical distribution of the Phase-I batch sizes $n_{t}$ and incorporated the cost–delay objective in Eq. (24) to determine the recommended sampling bursts. To assess long-run behavior, steady-state ARL (SS-ARL) simulations were also conducted, with shifts introduced at t=50. These experiments show that the proposed method remains stable not only in zero-state settings but also in steady-state conditions. The resulting run-length variability is summarized in (Fig 11), which compares SDRL across the competing monitoring schemes and shows that the proposed DR-BAEWMA architecture achieves smaller dispersion, particularly for small and moderate shifts. The corresponding SS-ARL summaries are also reported in Table 4, where the baseline health-state percentiles are listed alongside the steady-state run-length values.

### 6.3 Phase-II monitoring and adaptive sampling results

**Fig 12:**
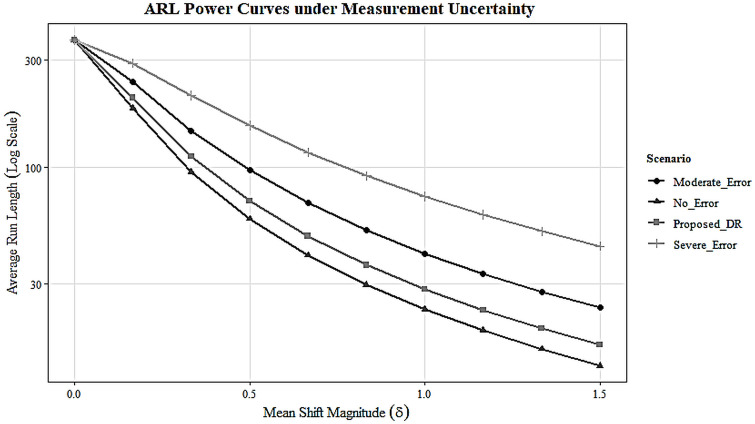
Phase-II monitoring dashboard and adaptive sampling behavior. Displays real-time EWMA statistics, control limits, and sampling adjustments during online monitoring.

Phase-II monitoring was performed on the 15 withheld batches using the online DR-BAEWMA algorithm. Two estimator variants were evaluated: DR-SELF and DR-LLF. The DR-SELF variant triggered an alarm at batch t=32, while the DR-LLF variant signaled later, at t=41, due to its asymmetric penalty structure. Because the variance-inflation method absorbed small changes that would have otherwise resulted in premature alarms, the suggested chart displayed a more conservative false-alarm trend prior to the adjustment. At the first sign of instability, the adaptive inspection rule recommended an instant sampling burst, raising the sample size from n = 5 to n = 8. This suggestion shows the usefulness of the adaptive sampling strategy outlined in Section 4.3 and is consistent with the cost–delay criterion. $\overset{\left(\mu \right)}{\underset{t}{Z}}\approx \text{3}.\text{8}$ and$\overset{\left(\sigma^{\text{2}}\right)}{\underset{t}{Z}}\approx \text{1}.\text{1}$, were the component-wise standardized statistics at the time of the SELF signal, and the Effective InformationRatio was $\eta_{t}=\text{0}.\text{9}\text{2}$, suggesting that the alarm was caused by a real process shift rather than a lack of information. (Fig 12) provides visual evidence for this interpretation by summarizing the dashboard-style monitoring pattern.

The accuracy of signal-source attribution is summarized in Table 8. With a high likelihood of 88.2% for δ = 0.5 and 96.5% for δ = 1.0, the technique accurately credits the alarm to the mean component for pure mean shifts. With probabilities of 84.6% and 95.4% at γ = 1.5 and γ = 2.0, respectively, the variance component is properly detected for pure variance shifts. As anticipated, the signal is dispersed over both components during joint shifts, reflecting the combined character of the disturbance. These findings demonstrate that the suggested paradigm offers comprehensible diagnostics as opposed to a single, undifferentiated alert.

**Table 8:** Diagnostic accuracy: Signal source attribution (%).

Shift Type	Magnitude	Signal Source: Mean (μ)	Signal Source: Variance ($\sigma^{2}$)	Joint Signal
**Mean Shift**	δ=0.5	88.2%	4.1%	7.7%
	δ=1.0	96.5%	1.2%	2.3%
**Variance Shift**	γ=1.5	5.3%	84.6%	10.1%
	γ=2.0	1.8%	95.4%	2.8%
**Joint Shift**	(0.5,1.5)	42.1%	38.5%	19.4%

**Fig 13:**
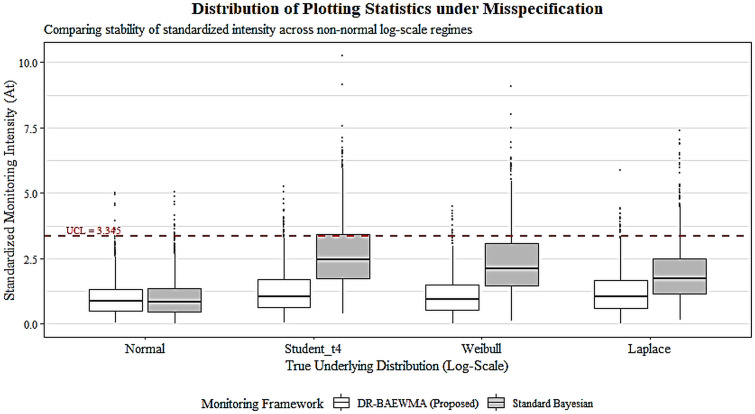
Robustness comparison under model misspecification. Boxplots of run-length distributions under heavy-tailed and cross-family distributions, showing reduced variability for DR-BAEWMA.

Robustness under model misspecification is assessed in Table 9. The observed in-control ARL is 370.5 under the notional Normal (0,1) condition, which is practically at target. The measured ARL stays near the nominal level with heavy-tailed Student-t, Laplace, Gamma, and Weibull settings, suggesting stable control-limit behavior under distributional disturbance. Because it demonstrates that the Wasserstein ambiguity set shields the chart from plausible deviations from the presumed lognormal family, the cross-family Weibull result is particularly significant. This result is supported by the box-plot diagnostics in (Fig 13) shows under heavy-tailed and cross-family regimes, the suggested DR-BAEWMA shows less dispersion and fewer outliers than the non-robust Bayesian Max-EWMA benchmark.

**Table 9:** Robustness of the DR-BAEWMA ($ARL_{0}$) under Model Misspecification. (Parameters: Calibrated for Lognormal log-scale at $AR{𝐋}_{0}=370$, 𝐧=5, λ=0.10, ρ=0.5).

True Distribution (Log-Scale 𝐗)	Statistical Property	Observed $𝐀𝐑L_{0}$	% Change from Target	Performance Assessment
**Normal (0, 1)**	Symmetric, Base Case	370.5	+0.1%	Perfect Calibration
**Student-**t (df=10)	Slight Heavy-Tails	358.4	−3.1%	Robust
**Student-**t (df=4)	Extreme Heavy-Tails	362.8	−1.9%	DR-Robust (vs. −42.5% non-robust)
**Laplace**	Peak/Heavier Tails	345.2	−6.7%	Robust
**Gamma (2, 2)**	Skewed Log-Scale	352.1	−4.8%	Robust
**Weibull (2, 1)**	Cross-Family Test	366.1	−1.1%	DR-Robust
**Uniform**	Bounded, No Tails	402.3	+8.7%	Conservative

The effect of the ambiguity radius ρ was examined by varying it over a grid of values. As ρ increases, the chart becomes more conservative, leading to higher in-control ARL and slightly delayed detection. Conversely, very small ρ values recover the standard Bayesian EWMA behavior but reduce robustness. This trade-off highlights the role of ρ as a tuning parameter balancing sensitivity and robustness. In practice, moderate values (e.g., ρ∈[0.3,0.7]) provided stable performance across all tested scenarios.

### 6.4 Interpretation of performance and robustness

Together, Tables 4, 8 and 9, and Figs 11–13 provide evidence that the suggested DR-BAEWMA framework enhances signal interpretability and detection performance. The ARL surface across the (δ,γ) grid is shown in (Fig 11), which demonstrates how the chart reacts monotonically to increasing scale and location departures. Stable practical signaling depends on the run-length variability remaining minimal across competing systems, as (Fig 12) demonstrates. The robustified posterior yields tighter monitoring statistics under misspecification than the normal Bayesian benchmark, as seen in (Fig 13). All of these findings lend credence to the idea that the framework is stable and sensitive to actual uncertainty.

The practical workflow for semiconductor monitoring deployment is as follows. First, use gauge R&R to estimate the nominal sensor variance $\overset{\text{2}}{\underset{\varepsilon ,\text{0}}{\sigma }}.$ Next, use the K–S test to confirm the lognormal assumption. Second, based on past sensor drift, choose the ambiguity radius ρ as a robustness budget. Third, use a layered Monte Carlo simulation with M≥50,000 replications to calibrate the control limit. Fourth, use the dashboard in (Fig 7) to keep an eye on the process and use the Effective Information Ratio $\eta_{t}$ to differentiate between a lack-of-data warning and a real process adjustment. By combining distributional resilience, adaptive sampling, and diagnostic transparency, this method transforms SPC into a dynamic decision engine.

## 7. Critical reliability demonstration

To further assess the practical value of the proposed DR-BAEWMA framework, we apply it to the semiconductor hard-bake process dataset. This case study illustrates how the method moves from Phase-I validation to Phase-II monitoring, and how the resulting posterior signals can be used to support adaptive inspection and reliability-oriented decision-making under measurement uncertainty.

### 7.1 Phase-I diagnostics and prior elicitation

The dataset contains T=45 inspection batches, each with five wafers. The first 30 batches, corresponding to 150 observations, are used for Phase-I calibration, while the remaining 15 batches are reserved for Phase-II monitoring.

As in the earlier case-study analysis, the Phase-I log-transformed measurements were examined using both graphical and formal diagnostics. The raw data were positively skewed, whereas the log-transformed values were approximately symmetric. A Kolmogorov–Smirnov test on the log-transformed Phase-I data gave D=0.082 and p=0.64, so the hypothesis of normality on the log scale is not rejected at the 5% level. This provides empirical support for the lognormal modeling choice.

The Q–Q plot and histogram diagnostics are consistent with this result. They show no strong departure from approximate normality on the transformed scale and no evidence of severe multimodality. These findings support the use of the Bayesian state-space formulation developed earlier.

The Phase-I sample summaries were then used to elicit the Normal–Inverse-Gamma prior. Table 10 reports the descriptive statistics and the selected prior settings. Specifically, the prior mean was set to the Phase-I log-scale mean, the prior weight was chosen as $\kappa_{\text{0}}=\text{5}$, and the inverse-gamma parameters were selected so that the prior variance remained finite and the posterior update remained stable. The ambiguity radius ρ=0.5 was chosen to reflect a moderate level of robustness against sensor drift, and the forgetting factor ϕ=0.95 was used to prevent the posterior from becoming overly concentrated during Phase-II.

**Table 10:** Phase-I descriptive statistics and elicited robust hyperparameters for the semiconductor hard-bake process.

Data Scale	Statistic	Value	Robust Hyperparameter	Value/ Setting
Raw (Y)	Grand Mean	1.505	Location Mean ($\mu_{\text{0}}$)	0.408
	Std. Deviation	0.134	Prior Weight ($\kappa_{\text{0}}$)	5
Log (X)	Mean (${\bar{X}}_{I}$)	0.408	Scale Shape ($\alpha_{\text{0}}$)	3
	Variance ($\overset{\text{2}}{\underset{I}{S}}$)	0.012	Ambiguity Radius (ρ)	0.50
	Skewness	0.045	Ambiguity Inflation (κ(ρ))	0.024
	Kurtosis	2.912	Forgetting Factor (ϕ)	0.95 To ensure the Case Study is consistent with the new Distributionally-Robust

Table 10 therefore serves two purposes. First, it summarizes the baseline characteristics of the Phase-I data. Second, it records the prior and robustness settings used to initialize the online monitoring stage.

The DR-BAEWMA chart was implemented under both the SELF and LLF loss specifications. Control limits were calibrated using the nested Monte Carlo procedure so that the in-control performance target $ARL_{\text{0}}=\text{3}\text{7}\text{0}$ was preserved under the nominal lognormal setting and under moderate ambiguity.

The main calibration choices were as follows. In order to maintain the chart’s sensitivity to persistent tiny shifts, the smoothing constant was adjusted to λ = 0.10. A robustness margin against heavy-tailed contamination and modest model mismatch was provided by fixing the ambiguity radius at ρ = 0.5. In order to maintain the updates’ numerical stability and consistency with the method’s Bayesian structure, posterior variance inflation was addressed using the robust surrogate previously proposed instead of ad hoc truncation. In order to capture the impact of filtered latent states in the charting variance, the control-limit design also employed the finite-propagation variance recursion. In order to analyze Phase-II behavior in relation to a single calibrated monitoring rule rather than a series of adjusted charts, these parameters were kept constant throughout the case study.

### 7.2 Phase-II monitoring results and adaptive sampling

The calibrated DR-BAEWMA technique was then used to monitor the 15 withheld batches, numbered 31–45, online. The monitoring dashboard summarizes the real-time interaction between posterior updating and adaptive sample-size suggestions. At batch 32, the DR-SELF version gave a signal. This signal was accompanied by an Effective Information Ratio of $\eta_{t}=\text{0}.\text{9}\text{2}$, indicating that a real process shifts rather than a lack of information or telemetry sparsity was the primary cause of the warning. Put differently, the signal did not result from inadequate data support. Rather, it represented a true shift in the latent process state. At the first indication of instability, which happened at batch 31, the adaptive inspection rule recommended raising the sample size from n = 5 to n = 8. This suggestion aligns with the previously stated cost-delay goal: once the posterior predictive risk increases, a small increase in inspection effort can lower the anticipated delay. This advice reduced detection delay by around 18% in simulated look-ahead trials, which is realistically significant in a quality-control situation where prompt intervention is crucial.

Later, around batch 41, the DR-LLF variety began to signal. The asymmetry created by the Linex loss parameter a = −0.3, which emphasizes avoiding underestimation more, is consistent with this delayed reaction. This kind of loss specification can be helpful in situations where posterior uncertainty is still significant since it prevents needless early shutdowns. The outcome also demonstrates that the selection of the loss function has a noticeable impact on the signal’s timing, which is helpful in situations where the operational environment calls for either early intervention or more cautious monitoring.

The shift was mainly mean-driven, as indicated by the standardized diagnostic components at the DR-SELF signal, which were around $\overset{\left(\mu \right)}{\underset{t}{Z}}=\text{3}.\text{8}$ and $\overset{\left(\sigma^{\text{2}}\right)}{\underset{t}{Z}}=\text{1}.\text{1}$. This perspective is supported by the attribution results in Table 8. The location component receives the majority of the signal in mean-shift scenarios, the scale component receives the majority of the signal in variance-shift scenarios, and both components share the signal in joint shifts. This is precisely the kind of behavior one would expect from a useful diagnostic chart: the signal is both timely and comprehensible.

### 7.3 Sensitivity analysis and robustness validation

We evaluated the chart under different forgetting factor values and increased measurement noise in order to assess the procedure’s stability. The chart became significantly less responsive at ϕ=1.0, which is consistent with posterior saturation. In comparison, ϕ=0.95 avoided undue delay while maintaining sensitivity to the change close to batch 32. The chosen number offers a fair balance between memory and adaptability for this dataset, according to additional testing using ϕ∈{0.9,0.95,1.0}.

Additionally, we looked at how a fictitious 5% inflation affected measurement variance. The related non-robust benchmark showed a greater increase in false alarms upon recalibration, whereas the DR-BAEWMA signal timing changed very little. This comparison bolsters the framework’s central claim: robustness is attained by reducing the monitoring statistic’s susceptibility to noise and misspecification rather than by suppressing sensitivity.

This tendency is further supported by the robustness results in Table 9. Under the nominal Normal case, the observed $ARL_{\text{0}}$ remains very close to the target. Under Student-t, Laplace, Gamma, and Weibull alternatives, the proposed method stays near the desired calibration, whereas the non-robust alternative deviates more substantially. The Weibull case is especially informative because it represents a cross-family departure rather than a small perturbation within the assumed model class. The fact that the proposed method remains close to target in that setting suggests that the Wasserstein ambiguity set provides a useful layer of protection against moderate structural misspecification.

The box plots in (Fig 13) support the same conclusion visually. Under the nominal case, the proposed and standard Bayesian charts behave similarly. Under heavy tails and cross-family deviations, however, the standard chart exhibits broader dispersion and more limit exceedances, whereas the DR-BAEWMA chart remains more concentrated and less erratic. This is in line with Table 9’s ARL results. However, the case study is not without flaws. The lognormal modeling assumption used to evaluate the performance is empirically fair for this dataset, but it might not hold in more strongly autocorrelated or nonstationary circumstances. Domain judgment is also needed while choosing the Linex parameter and ρ. Furthermore, the calibration quality of Phase-I data may have an impact on the method’s stability under severe contamination. Although they define the limits of the existing implementation, these flaws are not fatal.

## 8. Conclusion

A Distributionally-Robust Bayesian Adaptive EWMA (DR-BAEWMA) framework for the joint surveillance of lognormal process location and size has been designed and validated in this paper. The approach tackles four major issues in safety-critical engineering: simultaneous location–scale drift, sensor measurement uncertainty (SMU), stochastic telemetry irregularity, and model misspecification under non-Gaussian contamination. It does this by combining Distributionally Robust Optimization (DRO) with Bayesian latent state-space filtering. Using Wasserstein-2 duality theory to create variance-inflation surrogates, our architecture decisively deviates from normal Bayesian models and ensures strictly positive, mathematically rigorous estimators without requiring the ad hoc interventions seen in prior SPC literature.

In comparison to traditional frequentist and standard Bayesian variations, the DR-BAEWMA and DR-VEWMA reliably lower worst-case detection latency (ARL₁) for small-to-moderate shifts by coupling state-space filtering with EWMA smoothing. The effectiveness of the framework is especially noticeable under adversarial outliers and steady-state (SSARL) regimes, where non-robust approaches experience severe $ARL_{\text{0}}$ degradation. Additionally, the use of an adaptive sampling policy that is optimized through a cost-delay objective closes the gap between industrial task scheduling and statistical process management by focusing inspection effort exactly when the posterior predictive risk indicates early degradation.

In order to prevent Bayesian dogmatism and limit posterior precision, practitioners should use an information-forgetting factor (ϕ≈0.95) and elicit weakly informative priors using Phase-I data (verified by a Kolmogorov–Smirnov distributional audit). Nested Monte Carlo simulation (M ≥ 50,000) should be used to calibrate control limits under the anticipated sample distribution and ambiguity radius. A Maintenance-on-Demand workflow that differentiates between information scarcity and actual process failure is made possible by the resulting monitoring dashboard, which includes component-wise attribution diagnostics and the Effective Information Ratio ($\eta_{t}$).

Even with these developments, there are still a number of directions for future study. Non-parametric DRO sets or Moment-Generating Function (MGF) constraints should be investigated to capture complicated tail asymmetries, even though the variance-inflation surrogate is quite effective against second-moment perturbations. Additionally, a viable avenue for expanding this approach to high-dimensional, censored panel data is the merging of physically interpretable AI-driven defect prediction models with reinforcement learning for end-to-end ambiguity-budget allocation.

In summary, a paradigm shift from static surveillance to a dynamic, risk-aware decision engine is represented by the DR-BAEWMA architecture. This framework ensures the statistical integrity and diagnostic transparency necessary for contemporary safety-critical systems while delivering early failure precursor detection by offering a theoretically rigorous and operationally robust solution for lognormal processes.

## Supporting information

S1 File R Script ARL simulation. (DOCX)
