# Supplementary material for: A distributionally-robust bayesian adaptive EWMA chart for joint surveillance of lognormal process location and scale
Source: PLoS One. 2026 Jul 9;21(7):e0343029. doi: 10.1371/journal.pone.0343029 (PMC13349310; doi:10.1371/journal.pone.0343029)
Supplement: S1 File — (DOCX) [file pone.0343029.s001.docx]

# DR-BAEWMA SIMULATION (Lognormal Process Monitoring)

# ------------------------------------------------------------------------------

# This script simulates a Bayesian EWMA monitoring scheme with:

# - Robust Bayesian updating

# - EWMA tracking of mean and variance

# - Monte Carlo estimation of run length (ARL)

# ==============================================================================

library(dplyr)

library(ggplot2)

# ------------------------------------------------------------------------------

# 1. Helper function: variance inflation term

# ------------------------------------------------------------------------------

get_kappa <- function(rho, sigma_eps_0) {

# Simple inflation term for robustness adjustment

2 * sigma_eps_0 * rho + rho^2

}

# ------------------------------------------------------------------------------

# 2. Bayesian update step

# ------------------------------------------------------------------------------

dr_bayesian_update <- function(prev, batch, phi, rho, sigma_eps_0) {

n <- length(batch)

x_bar <- mean(batch)

ss <- sum((batch - x_bar)^2)

kappa_inf <- get_kappa(rho, sqrt(sigma_eps_0))

# update hyperparameters with forgetting factor

kappa_new <- phi * prev$kappa + n

alpha_new <- phi * prev$alpha + n / 2

mu_new <- (phi * prev$kappa * prev$mu + n * x_bar) / kappa_new

beta_new <- phi * prev$beta +

0.5 * ss +

(phi * prev$kappa * n * (x_bar - prev$mu)^2) / (2 * (phi * prev$kappa + n)) +

kappa_inf

list(

mu = mu_new,

kappa = kappa_new,

alpha = alpha_new,

beta = beta_new

)

}

# ------------------------------------------------------------------------------

# 3. ARL simulation function

# ------------------------------------------------------------------------------

simulate_arl <- function(

M = 5000,

lambda = 0.10,

H = 3.345,

phi = 0.95,

rho = 0.5,

sigma_eps_0 = 0.05,

steady_state = FALSE

) {

run_lengths <- numeric(M)

# initial values (Phase I)

mu0 <- 0

kappa0 <- 5

alpha0 <- 3

beta0 <- 0.024

for (m in 1:M) {

params <- list(mu = mu0, kappa = kappa0, alpha = alpha0, beta = beta0)

P <- mu0

Q <- beta0 / (alpha0 - 1)

t <- 0

alarm <- FALSE

warmup <- if (steady_state) 50 else 0

while (!alarm) {

t <- t + 1

# generate sample batch (log-domain)

batch <- rnorm(5, mean = 0, sd = 1)

# update Bayesian parameters

params <- dr_bayesian_update(params, batch, phi, rho, sigma_eps_0)

mu_hat <- params$mu

var_hat <- params$beta / (params$alpha - 1)

# EWMA update

P <- lambda * mu_hat + (1 - lambda) * P

Q <- lambda * var_hat + (1 - lambda) * Q

# standardized monitoring statistics (simulation scaling)

Z_mu <- abs(P - mu0) / 0.35

Z_var <- abs(Q - 1.0) / 0.55

statistic <- max(Z_mu, Z_var)

if (t > warmup && statistic > H) {

run_lengths[m] <- t - warmup

alarm <- TRUE

}

if (t > 10000) {

run_lengths[m] <- 10000

alarm <- TRUE

}

}

}

run_lengths

}

# ------------------------------------------------------------------------------

# 4. Run simulation

# ------------------------------------------------------------------------------

set.seed(2026)

cat("Running simulation...\n")

results <- simulate_arl(

M = 10000,

lambda = 0.10,

steady_state = TRUE

)

# summary table

summary_table <- data.frame(

Metric = "DR-BAEWMA",

P10 = quantile(results, 0.10),

P25 = quantile(results, 0.25),

Median = median(results),

P90 = quantile(results, 0.90),

ARL = mean(results)

)

print(summary_table)

# ------------------------------------------------------------------------------

# 5. Run length distribution plot

# ------------------------------------------------------------------------------

df <- data.frame(RunLength = results)

p <- ggplot(df, aes(x = RunLength)) +

geom_histogram(bins = 50, color = "white") +

theme_bw(base_family = "serif") +

labs(

title = "Run Length Distribution",

x = "Run Length",

y = "Frequency"

) +

geom_vline(xintercept = mean(results), linetype = "dashed")

# ggsave("run_length_distribution.tiff", p, width = 6, height = 4, dpi = 300)
