# Supplementary material for: A distributionally-robust bayesian adaptive EWMA chart for joint surveillance of lognormal process location and scale
Source: PLoS One. 2026 Jul 9;21(7):e0343029. doi: 10.1371/journal.pone.0343029 (PMC13349310; doi:10.1371/journal.pone.0343029)
Supplement: Attachment — Submitted filename: review.docx [file pone.0343029.s002.docx]

The paper proposes a Bayesian Max-EWMA framework for real-time monitoring of lognormal process mean and variance, accounting for measurement error and variable batch sizes, and demonstrates early detection of deviations and robustness in industrial applications. The topic is relevant and potentially valuable to the fields of statistical process control (SPC) and reliability monitoring.

However, in its current form, the manuscript suffers from significant scientific, methodological, validation, mathematical transparency, and presentation deficiencies. Several claims are made without formal justification or adequate comparative evaluation, and parts of the proposed methodology lack sufficient rigor or clear explanation.

**Major comments:**

1. The authors propose a Bayesian Max-EWMA framework for lognormal processes. However, Max-EWMA charts have been previously introduced, Bayesian EWMA charts are extensively studied, and the conjugate NIG model for normal mean–variance monitoring is standard. The manuscript should provide direct comparisons with state-of-the-art methods and include a table summarizing theoretical and practical contributions relative to existing studies. It should also clarify the role of key components, such as measurement-error correction, the Max statistic, and Linex loss integration.
2. The manuscript does not provide a clear and precise definition of the Baseline Health State model.
3. Please pay careful attention to the structure and writing of the manuscript. Several obvious writing errors are observed, and the paper requires a thorough revision.

Examples include:

Page 3, line 76: *“1.3 Joint monitoring and Max-type statistics Joint monitoring”* – it is unclear what “1.3” refers to.

Page 17, line 365: *“the per-Inspection Batch estimators at time 𝑡under the Baseline Health State model.”* – there is no space between “𝑡” and “under”. Similar errors appear elsewhere in the manuscript.

Page 21, line 458: *“The objective is to monitor for shifts in the underlying process parameters (𝜇,𝜎^2^)”* – the notation for the parameters is incorrect.

These are just a few examples of writing issues. Careful attention is required to correct such errors throughout the manuscript.

4. The study assumes that the observations $X_{it}$ are independent and identically distributed (i.i.d.) following a normal distribution with constant mean and variance. However, in reliability and degradation monitoring, data are often autocorrelated, time-dependent, and non-stationary. This assumption is highly restrictive and limits the applicability of the proposed method. The authors should justify it by examining the effect of autocorrelation, performing a robustness analysis, or explicitly acknowledging it as a limitation.
5. The Linex loss is essentially used in an ad hoc manner. The authors themselves acknowledge that for the student-t distribution, the exponential moment may not exist, and for $\sigma^{2}$, the integrals diverge. Moreover, the Linex loss has no role in error control or ARL: it only affects point estimation, with no theoretical analysis of how $a$ impacts ARL, no guidance on choosing $a$, and no robustness check against model misspecification. As a result, the Linex loss appears mostly decorative rather than effective, and its removal would likely have minimal impact on the results. The authors should clarify and justify why the Linex loss is included at all.
6. A serious concern is the negative estimates of $\sigma^{2}$. The paper itself states that “*if* $\beta_{t}/(\alpha_{t}-1)<\tau^{2}$ *occurs, the estimator becomes negative … enforce a floor*” which constitutes a direct violation of Bayesian principles, making the resulting estimator no longer truly Bayesian. This represents a significant weak spot in the proposed methodology.
7. The data are not publicly accessible. PLOS ONE places strong emphasis on Data Availability, yet the authors state that the data are available only “upon reasonable request.” This is generally not acceptable for PLOS ONE and limits reproducibility.
8. The definition of FEWMA is not clearly specified.

These issues should be addressed consistently throughout the paper.

9. Some subsection headings appear without numbering and are presented only in boldface, which disrupts the structural consistency of the manuscript. For example, *“Notation and Assumptions”, “Bayes Estimators under SELF”* and *“Variance of the Dispersion Estimator”* are not numbered like other sections. Please ensure that all sections and subsections are consistently numbered throughout the manuscript.
10. Several phrases and subsection titles are repeated verbatim in the manuscript. For example, *“Bayes Estimators under LLF: Bayes Estimators under LLF”* appears consecutively in duplicate form. Such repetitions suggest insufficient proofreading and affect the overall presentation quality. A thorough and careful revision of the entire manuscript is strongly recommended to eliminate redundancies and improve clarity and consistency.
11. Many sentences, particularly in the Introduction and Methodology sections, are excessively long and contain multiple ideas in a single sentence, which reduces clarity. The authors are encouraged to split such sentences into shorter, more focused statements.
12. The manuscript frequently capitalizes common technical terms (e.g., Baseline Health State, Inspection Batch, Sensor Measurement Uncertainty) that are not proper nouns. Capitalization should be used consistently and limited to proper names or formally defined acronyms.
13. Several statements use strong, promotional language (e.g., “offers a principled tool,” “ensures robustness”) without sufficient empirical or theoretical support. A more neutral, evidence-based scientific tone is recommended.
14. Verb tense usage is inconsistent across sections, particularly between present and past tense in the Methods and Simulation sections. The authors should standardize tense usage.
15. Several symbols and notations are introduced without immediate definition or are redefined later in the manuscript. All symbols should be clearly defined at their first occurrence and used consistently thereafter.
16. Some closely related concepts are described using different terms across sections (e.g., variability, dispersion, and volatility), which may confuse readers. The authors are encouraged to standardize terminology throughout the manuscript.
17. The manuscript contains scattered minor grammatical and typographical errors (e.g., missing articles, inconsistent hyphenation, spacing issues). A careful language edit is recommended to improve overall polish.
18. Figure and table captions should provide concise and clear titles that identify the content of the figure or table, rather than descriptive explanations. All figure and table captions should be carefully reviewed and revised accordingly.
19. Some equations in the manuscript are numbered while others are not. All mathematical expressions presented as displayed equations within the text should be consistently numbered.
20. Some figure references in the manuscript are incorrect. For example, the text states “is compared in Figure 10,” whereas the correct reference should be Figure 11. Please carefully check and correct all in-text references throughout the manuscript.
21. The references should be presented in a uniform and correct format.
22. It is recommended to provide a table listing all abbreviations and their corresponding full terms. Throughout the manuscript, consistently use the abbreviations instead of repeatedly writing long expressions.
