# Supplementary material for: A distributionally-robust bayesian adaptive EWMA chart for joint surveillance of lognormal process location and scale
Source: PLoS One. 2026 Jul 9;21(7):e0343029. doi: 10.1371/journal.pone.0343029 (PMC13349310; doi:10.1371/journal.pone.0343029)
Supplement: Attachment — Submitted filename: Point by point response to the reviewers comments.docx [file pone.0343029.s004.docx]

Response to Reviewers

Manuscript ID: PONE-D-26-04764

**Title (Revised): *A Distributionally-Robust Bayesian Adaptive EWMA Chart for Joint Surveillance of Lognormal Process Location and Scale***

We sincerely thank the Academic Editor and the reviewers for their careful evaluation of our manuscript and for the constructive comments that have helped us improve both the clarity and the technical depth of the work. The original submission, titled *“A Bayesian Max-EWMA Chart for Joint Surveillance of Lognormal Process Location and Scale,”* presented a Bayesian extension of the Max-EWMA framework. However, in light of the reviewers’ concerns, particularly regarding novelty, robustness, and methodological rigor, we have undertaken a substantial revision of the manuscript.

In the revised version, we introduce a ***Distributionally-Robust Bayesian Adaptive EWMA (DR-BAEWMA*)** framework. This is not a superficial modification, but a meaningful extension that incorporates distributional robustness, adaptive state-space modeling, and a more principled treatment of uncertainty. These changes address the key concerns raised by the reviewers and significantly strengthen the contribution of the paper.

We have revised the manuscript thoroughly and respond to each comment in detail below.

***Editor Comment 1:*** *Compliance with PLOS ONE style requirements*

Response:
We have carefully revised the manuscript to ensure full compliance with PLOS ONE formatting and style guidelines. Specifically, All section headings and subheadings have been consistently numbered. Figure and table captions have been standardized to concise, descriptive titles. Notation and formatting inconsistencies have been corrected throughout the manuscript. File naming conventions and manuscript structure have been aligned with the journal’s requirements.

***Editor Comment 2:*** *Code availability and reproducibility*

Response:
We fully agree with the importance of reproducibility. In the revised manuscript, a complete reproducibility workflow has been added in Sections 6 and 7. The manuscript now clearly describes the Data preprocessing (Phase-I and Phase-II separation), Model calibration via nested Monte Carlo simulation and online monitoring implementation. The R code used for simulations and case study analysis has been prepared and will be provided as Supplementary Material, with detailed comments to facilitate reuse and verification.

***Editor Comment 3:*** *Data availability statement revision*

Response:
We have revised the Data Availability Statement to comply with PLOS ONE policy.

***Editor Comment 4****: Missing in-text reference to Table 6*

Response:
Thank you for noting this. We have corrected this oversight; Table 6 is now explicitly referenced in the text (Sections 6 and 7). The discussion surrounding Table 6 has been expanded to clarify its role in signal-source attribution and interpretability.

Response to Reviewer #1

We thank Reviewer #1 for the positive evaluation of our work and for the constructive suggestions to further strengthen the manuscript. All comments have been carefully addressed, as detailed below.

***Comment 1****: To further strengthen the context and breadth of your contribution, I recommend engaging with recent advancements in related fields. Specifically, the work would benefit from a discussion with studies focusing on risk-informed decision-making for critical infrastructure maintenance, advanced metaheuristic optimization techniques applied to real-world scheduling challenges, and the latest trends in AI-driven non-destructive evaluation and quality control in manufacturing using deep learning for defect detection. Additionally, incorporating insights from recent data-driven process modeling in metallurgy and materials science could provide valuable perspectives on generalizing your current findings or contrasting your methodology with established industrial prediction approaches. You can cite the following works:*

*• Zhou, N., Luo, L., Sheng, G., & Jiang, X. (2025). Scheduling the Imperfect Maintenance and Replacement of Power Substation Equipment: A Risk-Based Optimization Model. IEEE Transactions on Power Delivery, 40(4), 2154-2166. doi: 10.1109/TPWRD.2025.3572076*

*• Long, X., Cai, W., Yang, L., & Huang, H. (2024). Improved particle swarm optimization with reverse learning and neighbor adjustment for space surveillance network task scheduling. Swarm and Evolutionary Computation, 85, 101482. doi: https://doi.org/10.1016/j.swevo.2024.101482*

*• Xu, H., Han, F., Zhou, W., Liu, Y., Ding, F.,... Zhu, J. (2024). ESMNet: An enhanced YOLOv7-based approach to detect surface defects in precision metal workpieces. Measurement, 235, 114970. doi:https://doi.org/10.1016/j.measurement.2024.114970*

*• Xia, Y., Song, Q., Yi, B., Lyu, T., Sun, Z.,... Li, Y. (2025). Improving out-of-distribution generalization for online weld expulsion inspection using physics-informed neural networks. Welding in the World, 69(5),1309-1322. doi: 10.1007/s40194-025-01950-6*

*• Wang, G., Yang, Y., Zhou, S., Li, B., Wei, Y.,... Wang, H. (2024). Data Analysis and Prediction Model for Copper Matte Smelting Process. Metallurgical and Materials Transactions B, 55(4), 2552-2567. doi:10.1007/s11663-024-03115-0*

Response:
We appreciate this insightful suggestion. To strengthen the contextual positioning of our work, the introduction has been expanded to incorporate recent developments in risk-informed decision-making for infrastructure maintenance, metaheuristic optimization in scheduling, AI-driven defect detection and non-destructive evaluation and Data-driven modeling in metallurgy and materials science. The suggested references have been carefully reviewed and incorporated where relevant.

***Comment 2:*** *Improve figure formatting (fonts, labels, etc.)*

Response:
We have revised all figures to improve clarity and consistency: Fonts (including axis labels and tick marks) have been standardized to Times New Roman, as recommended. Figure layouts have been refined for readability. All graphical elements now follow a uniform style consistent with journal expectations.

**Comment 3:** *Add a literature review table*

Response:
We agree that a structured comparison enhances clarity. A comprehensive literature review table (Table 1) has been added. This table contrasts: Existing monitoring frameworks (Max-EWMA, Bayesian EWMA, AI-based methods, etc.). Their assumptions, robustness properties, and limitations, and The specific gap addressed by the proposed DR-BAEWMA framework

This addition strengthens the positioning and clarity of contributions.

***Comment 4****: The simulation section is well designed and implemented; no major revisions are required. My only suggestion is to highlight (e.g., bold) the best-performing method in each comparison table to enhance readability and understanding.*

Response:
Thank you for this practical suggestion. All simulation result tables have been revised to highlight the best-performing methods (e.g., lowest ARL values) using bold formatting. This significantly improves readability and interpretability of the results.

**Comment 5:** *Case study requires further development (data availability, step-by-step procedure, K–S test, EDA, flowchart)*

Response:
We have substantially strengthened the case study section (Sections 6 and 7) to address this comment: Data Validation (K–S Test): A formal Kolmogorov–Smirnov test is now included to justify the lognormal assumption. Results are reported alongside graphical diagnostics. Exploratory Data Analysis (EDA): A comprehensive diagnostic figure (Figure 7) has been added, including: Time-series plots, Histograms (raw and log-transformed), Q–Q plots, Autocorrelation analysis. These revisions significantly enhance the clarity, transparency, and reproducibility of the case study.

*Comment 6: Provide R/Python code for simulations*

Response:
We fully agree with this recommendation. The simulation and implementation code has been, organized and documented with explanatory comments. It will be provided as Supplementary Material, ensuring full reproducibility of simulation experiments, case study analysis, and control-limit calibration

***Comment:*** *The simulation section is well designed; highlight best-performing methods in tables*

Response:
We appreciate the reviewer’s positive assessment of the simulation design. To improve interpretability and readability, all simulation tables (now presented in Tables 4 and 5) have been revised to explicitly highlight the best-performing methods using bold formatting. The performance comparison is now more transparent, allowing readers to quickly identify minimum ARL values for out-of-control scenarios, and stability of in-control performance. These changes enhance the accessibility and clarity of the simulation results without altering their underlying structure.

***Comment:*** *The case study requires further development (dataset availability, step-by-step procedure, K–S test, EDA, flowchart, reproducibility)*

Response:
We fully agree with the reviewer that the case study should serve as a transparent and reproducible demonstration of the proposed methodology. Accordingly, Sections 6 and 7 have been substantially revised and expanded. The key improvements are summarized below:

(a) Dataset transparency and availability

The dataset description has been clarified, including structure of inspection batches, and measurement context and preprocessing steps. The Data Availability Statement has been revised in accordance with journal policy.

(b) Step-by-step implementation procedure

A structured workflow has been introduced to guide replication, covering phase-I data selection and baseline estimation, prior hyperparameter elicitation, calibration of control limits via nested simulation, online monitoring using DR-BAEWMA, and Interpretation of signals and adaptive decisions. This procedure is described explicitly in the text and visually summarized in Figure 9.

(c) Goodness-of-fit validation (K–S test)

A formal Kolmogorov–Smirnov (K–S) test has been incorporated to validate the lognormal modeling assumption. The results confirm that the log-transformed data are consistent with normality. The graphical comparison between empirical and fitted distributions is provided in Figure 8, supporting the formal test.

(d) Exploratory Data Analysis (EDA)

A comprehensive EDA has been added, including time-series visualization, histograms (raw and log-transformed data), Q–Q plot for normality assessment, and Autocorrelation function (ACF). These diagnostics are integrated into a single multi-panel figure (Figure 7) and discussed in Section 7.1. The EDA provides both model validation and insight into process dynamics, addressing the reviewer’s concern.

(e) Flowchart for practical implementation

A workflow diagram (Figure 9) has been added to illustrate the full implementation pipeline.

This includes data preprocessing, prior specification, robust calibration, and monitoring and decision-making. The flowchart enhances the practical usability of the proposed framework.

(f) Reproducibility (code and implementation)

The computational procedures used in simulation experiments, control-limit calibration, case study analysis have been clearly documented. The R/Python code is prepared and will be provided as Supplementary Material, including detailed comments, modular structure for reuse, and reproducible scripts for all reported results

***Comment:*** *Include sufficient explanations in code to help replication*

Response:
We fully agree with the importance of clear and well-documented code. All scripts have been revised to include inline comments explaining each step, clear labeling of inputs, parameters, and outputs and Modular functions for simulation generation, posterior updating, and control chart computation. A brief usage guide is also included in the supplementary material to assist readers in reproducing the results. This ensures that the computational aspects of the study are fully transparent and accessible.

Response to Reviewer #2

We thank the reviewer for the thoughtful and technically grounded comments. They go directly to the positioning and depth of the contribution, and addressing them has helped us sharpen both the methodology and its presentation. Below, we respond point-by-point.

**Comment 1:** *Comparison with existing work:*

*The following paper also proposes a MAX-EWMA approach:*

*“Joint monitoring of mean and variance using Max-EWMA for Weibull process” by Muhammad No2or-ul-Amin, Irfan Aslam, and Navid Feroze.*

*Please clarify how your work differs from this study. In that paper, two statistics are defined, whereas in your paper such distinction is not clearly presented. Elaborate on this difference to better position your contribution.*

Response: This is an important point, and we agree that the distinction needed to be made much clearer. In the revised manuscript, we now explicitly position our work relative to the Weibull-based MAX-EWMA study mentioned by the reviewer. The key difference is not just the underlying distribution, but the modeling philosophy and inferential structure. The existing Weibull MAX-EWMA approach is built in a classical SPC framework, where monitoring statistics are constructed directly from sample estimates of mean and variance (or their Weibull equivalents), typically under fixed distributional assumptions. In contrast, our revised framework, now formulated as Distributionally-Robust Bayesian Adaptive EWMA (DR-BAEWMA), operates within a Bayesian state-space setting, where the process parameters are treated as latent evolving states, inference is carried out via posterior updating rather than plug-in estimators, and uncertainty is explicitly modeled and propagated over time. More importantly, the current revision introduces distributional robustness via Wasserstein ambiguity sets, which fundamentally changes the problem. Instead of assuming the model is correctly specified (e.g., Weibull or lognormal), we allow for controlled deviations from the nominal model. The monitoring statistic is therefore calibrated under worst-case distributions within an ambiguity set, rather than a single assumed distribution.

Regarding the reviewer’s observation about “two statistics” in the existing work: In our framework, the separation between location and scale is preserved through standardized latent components $Z_{\mu}$and $Z_{\sigma}$, which are then combined through a Max-type aggregation. This is now explicitly clarified in the revised Section 4, where both components and their roles are formally defined and interpreted. To make this distinction transparent, we have added a comparative discussion in the Introduction and Methodology sections, and included a literature comparison table (Table 1) highlighting differences in inferential paradigm (classical vs Bayesian), treatment of uncertainty, robustness to misspecification, and adaptability and dynamic updating.

Overall, while both approaches fall under the broad umbrella of MAX-type EWMA monitoring, they address different statistical problems—ours being explicitly designed for robust, adaptive monitoring under model uncertainty.

**Comment 2:** **Choice of distribution:***The paper employs the lognormal distribution. However, the Weibull model is frequently used in reliability surveillance studies, while others sometimes adopt the lognormal model. Please justify your selection of the lognormal distribution and consider conducting a goodness-of-fit test—such as the Kolmogorov–Smirnov (K–S) test—to validate this choice.*

Response: We appreciate this comment and agree that the choice of distribution must be justified empirically rather than assumed. In the revised manuscript, we have strengthened this aspect in two ways. (1) The empirical justification via goodness-of-fit testing a formal Kolmogorov–Smirnov (K–S) test has been conducted on the log-transformed Phase-I data. The test results indicate no significant deviation from normality on the log scale, supporting the lognormal assumption for the original data. This result is now reported explicitly in Section 7.1, with graphical support provided in Figure 8. (2) We complement the formal test with a set of exploratory diagnostics (Figure 7), including histograms, Q–Q plots, time-series visualization, and autocorrelation analysis. These provide a more complete picture of the data structure and validate the modeling choice beyond a single test statistic.

At a conceptual level, we would also like to clarify our intent that the lognormal model is used as a baseline working model, consistent with many industrial degradation and reliability contexts. However, the proposed DR-BAEWMA framework is not restricted to the lognormal case. Through the introduction of distributional robustness, the method remains stable even when the true data-generating process deviates from lognormality. This is further demonstrated in the robustness study (Table 7), where performance is evaluated under alternative distributions, including Weibull. Thus, the revision both justifies the chosen model empirically and relaxes reliance on it theoretically, which we believe addresses the reviewer’s concern in a principled way.

**Comment 3:** *Simulation type—include steady-state analysis in addition to zero-state ARL*

Response:
We fully agree with the reviewer that steady-state performance is essential for a complete evaluation of control chart behavior. Accordingly, the simulation study has been expanded to include steady-state ARL analysis, in addition to the original zero-state results, the revised simulation framework now evaluates the zero-state ARL (initial detection performance), and steady-state ARL (long-run monitoring behavior under persistent operation).

Steady-state results are presented both, numerically in Table 4, and graphically in Figure 6.

The results show that the proposed DR-BAEWMA maintains stable in-control performance, and

exhibits consistent and timely detection under sustained shifts, without excessive variability.

We have also added explanatory discussion to clarify the difference between zero-state and steady-state perspectives, and why both are necessary for a realistic assessment of monitoring schemes.

This extension significantly strengthens the completeness and practical relevance of the simulation study.

***Comment 4:*** *Model robustness under Weibull distribution should be evaluated*

Response:
We fully agree with the reviewer that robustness across alternative data-generating mechanisms is essential for assessing the practical reliability of any monitoring scheme.

In response, we have significantly strengthened the robustness analysis in the revised manuscript. The performance of the proposed DR-BAEWMA framework has now been evaluated under multiple distributional settings, including Normal, Student-t, Laplace, Gamma, and importantly Weibull distribution, as explicitly suggested by the reviewer. The results are summarized in Table 7, where we report comparative ARL performance under each distributional scenario.

From a statistical standpoint, the key observation is that, while classical EWMA-type methods show noticeable degradation under Weibull and heavy-tailed settings, and the proposed DR-BAEWMA maintains stable and near-nominal in-control performance, with controlled sensitivity loss in out-of-control regimes.

This robustness is not accidental; it stems from the core structure of the model. The Wasserstein distributional ambiguity set allows the model to account for deviations from the assumed likelihood. The Bayesian updating mechanism ensures that uncertainty is propagated rather than underestimated.

The adaptive EWMA smoothing prevents overreaction to distributional misspecification.

We have also added a dedicated discussion in Section 7.4 to clarify that the Weibull case is not treated as an isolated experiment, but as part of a broader stress-testing framework for distributional misspecification.

***Comment 5:*** *More discussions:I think the authors can provide some other directions for this work as it can be applied in other fields. Based on my knowledge, I suggest to provide the following topics and cite the suggested works:*

*- Application of MAXXEWMA in quantile based moder: Hao, R., & Yang, X. (2024). Multiple-output quantile regression neural network. Statistics and Computing,34(2), 89.doi: 10.1007/s11222-024-10408-6 and Li, L., Xia, Y., Ren, S., & Yang, X. (2025). Homogeneity Pursuit in the Functional-Coefficient Quantile Regression Model for Panel Data with Censored Data, 29(3), 323-348. doi:10.1515/snde-2023-0024*

*- Fault detection with the proposed method: Wang, H., Li, Y., Men, T., & Li, L. (2024). Physically Interpretable Wavelet-Guided Networks With DynamicFrequency Decomposition for Machine Intelligence Fault Prediction. IEEE Transactions on Systems, Man and Cybernetics: Systems, 54(8), 4863-4875. doi: 10.1109/TSMC.2024.3389068 and Wan, A., Zhang, F., Al-Bukhaiti, K., Cheng, X., Ji, X., Wang, J.,... Shan, T. (2025). A Novel GA-PSO-SVM Model for Compound Fault Diagnosis in Gearboxes With Limited Data. IEEE Sensors Journal, 25(16),30443-30431.doi: 10.1109/JSEN.2025.3576761 and Lu, Y., Wang, S., Zhang, C., Chen, R., Dui, H., Mazurkiewicz, D.,... Zhang, Y. (2025). A dynamic imperfect inspection-based maintenance optimization considering dependent competing failure. Measurement, 253,117470. doi: https://doi.org/10.1016/j.measurement.2025.117470)*

Response:
We appreciate the reviewer’s suggestion to broaden the discussion and connect the methodology to emerging research directions. We agree that this strengthens the relevance of the work beyond statistical process control.

In the revised manuscript, we have expanded the Discussion section to include three main application perspectives.

(a) Extension to quantile-based monitoring frameworks: The reviewer’s suggestion regarding quantile regression and functional coefficient models is highly relevant.

We now clarify that the DR-BAEWMA framework can naturally be extended to quantile-driven monitoring statistics, where instead of tracking mean and variance, one monitors conditional quantiles of the process distribution, and tail-risk behavior under asymmetric loss structures.

This is particularly aligned with the distributionally robust formulation, since Wasserstein ambiguity sets are closely connected to worst-case expectations over neighborhoods of probability measures, which naturally extends to quantile-based risk measures.

We have incorporated the suggested works to highlight this connection, particularly in the context of multiple-output quantile regression, and censored functional coefficient models.

These references are now discussed to position the method within the broader robust predictive inference literature.

(b) Fault detection and machine learning integration

We also agree that modern fault detection systems increasingly rely on hybrid statistical–learning frameworks.

We now explicitly discuss how DR-BAEWMA relates to deep learning-based fault detection systems, Physics-informed neural networks for industrial monitoring, and data-driven diagnostic systems under limited or noisy observations.

From a statistical perspective, our contribution differs in an important way. The Machine learning models typically focus on prediction accuracy, often requiring large datasets. DR-BAEWMA focuses on sequential decision-making under uncertainty, where data arrive in real time, false alarms and detection delays have explicit cost interpretations, uncertainty is modeled explicitly rather than learned implicitly. We emphasize that the proposed method can complement machine learning systems by serving as a statistical monitoring layer on top of learned representations.

(c) Maintenance optimization and reliability systems

We have also expanded the discussion on reliability engineering applications, particularly, risk-based maintenance scheduling, dynamic inspection policies, and degradation-informed decision systems.

In this context, the DR-BAEWMA framework provides a real-time degradation indicator, and a statistically principled trigger mechanism for maintenance actions.

We highlight that the adaptive nature of the method makes it particularly suitable for non-stationary industrial environments, systems with evolving failure mechanisms, and partially observed degradation processes.

(d) Incorporation of suggested references

We have carefully reviewed all suggested references and included them where they are methodologically relevant. Specifically, works on quantile regression and functional coefficient models have been cited to support the extension to distributional risk monitoring. Fault detection and hybrid AI-based monitoring studies have been included to contextualize the modern industrial relevance of the proposed approach. Maintenance optimization literature has been incorporated to highlight decision-theoretic extensions of the framework.

Importantly, these citations are not added as superficial extensions, but are integrated into the narrative to clarify where DR-BAEWMA fits within the evolving ecosystem of robust statistical learning and industrial decision systems.

Response to Reviewer #3

We thank the reviewer for the detailed and technically rigorous evaluation. The concerns raised are substantive and have guided a major strengthening of the manuscript, both methodologically and in presentation. In particular, the paper has been substantially revised and extended into a Distributionally-Robust Bayesian Adaptive EWMA (DR-BAEWMA) framework to address issues of novelty, rigor, and robustness.

***Comment 1:*** *The authors propose a Bayesian Max-EWMA framework for lognormal processes. However, Max-EWMA charts have been previously introduced, Bayesian EWMA charts are extensively studied, and the conjugate NIG model for normal mean–variance monitoring is standard. The manuscript should provide direct comparisons with state-of-the-art methods and include a table summarizing theoretical and practical contributions relative to existing studies. It should also clarify the role of key components, such as measurement-error correction, the Max statistic, and Linex loss integration*

Response:
We appreciate this important observation. In the original version, the contribution was not positioned with sufficient clarity relative to existing work. This has now been comprehensively addressed. The manuscript has been fundamentally revised to introduce a Distributionally-Robust Bayesian Adaptive EWMA (DR-BAEWMA) framework, which differs from existing approaches in several key ways. Unlike classical Max-EWMA and standard Bayesian EWMA charts that rely on a fixed parametric model, the proposed method incorporates Wasserstein ambiguity sets, allowing inference and monitoring to remain valid under controlled deviations from the assumed distribution. Process parameters (location and scale) are modeled as time-evolving latent states, enabling adaptation to nonstationary environments. Measurement error, process variability, and model uncertainty are handled within a unified probabilistic framework, rather than through ad hoc corrections. The method includes a decision-theoretic component that adjusts inspection intensity based on current system state. These aspects collectively move the framework beyond a simple extension of Max-EWMA toward a robust, adaptive monitoring paradigm.

To address the reviewer’s concern, a comprehensive comparison table (Table 1) has been added, contrasting the proposed method with classical EWMA and Max-EWMA charts, bayesian EWMA approaches, and recent hybrid and data-driven monitoring methods

The comparison explicitly highlights differences in treatment of uncertainty, robustness to model misspecification, adaptability, and inferential structure.

The roles of the main components have now been clearly defined:

Measurement-error correction: Incorporated directly into the likelihood structure, allowing posterior updates to account for sensor uncertainty without biasing parameter estimates.

Max-type statistic: Constructed from standardized latent components $Z_{\mu}$and $Z_{\sigma}$, providing sensitivity to shifts in either location or scale while preserving interpretability.

Linex loss function: Used to model asymmetric risk preferences in estimation. Its role has now been clarified as influencing estimation behavior, not control chart calibration. Additional discussion has been added to explain when and why such asymmetry is relevant.

Overall, these revisions ensure that the novelty and contribution are now clearly articulated, technically grounded, and well positioned within the literature.

***Comment 2:*** *Baseline Health State model is not clearly defined*

Response:
We agree that the definition of the Baseline Health State (BHS) required clearer formalization.

In the revised manuscript. The BHS is now explicitly defined as the Phase-I posterior reference state, characterized by posterior mean vector for location and scale parameters, and associated uncertainty captured through the posterior covariance structure.

This definition is now introduced formally in the Methodology section and consistently used throughout the manuscript. We also clarify its operational role: It serves as the anchor for deviation detection in Phase-II monitoring, all monitoring statistics are constructed relative to this baseline.

This clarification ensures conceptual and mathematical consistency.

***Comment 3:*** *Please pay careful attention to the structure and writing of the manuscript. Several obvious writing errors are observed, and the paper requires a thorough revision.*

*Examples include:*

*Page 3, line 76: “1.3 Joint monitoring and Max-type statistics Joint monitoring” – it is unclear what “1.3” refers to.*

*Page 17, line 365: “the per-Inspection Batch estimators at time under the Baseline Health State model.” – there is no space between “ ” and “under”. Similar errors appear elsewhere in the manuscript.*

*Page 21, line 458: “The objective is to monitor for shifts in the underlying process parameters ( , 2)” – the notation for the parameters is incorrect.*

*These are just a few examples of writing issues. Careful attention is required to correct such errors throughout*

Response:
We appreciate the reviewer’s careful reading and fully agree that the manuscript required substantial improvement in presentation. Accordingly, the manuscript has undergone a thorough and systematic revision including structural consistency, clarity of writing, notation consistency, and typographical corrections. All sections and subsections are now properly numbered and aligned, redundant or duplicated headings have been removed. Long and complex sentences have been rewritten into clear, concise statements, transitions between sections have been improved to ensure logical flow. All symbols are now defined at first occurrence and used consistently. Ambiguous or inconsistent terminology has been standardized. Spacing, punctuation, and formatting issues have been corrected throughout. Errors such as incorrect equation notation and misplaced symbols have been fixed. These revisions substantially improve the readability, coherence, and professionalism of the manuscript.

***Comment 4****: The study assumes that the observations X_it are independent and identically distributed (i.i.d.) following a normal distribution with constant mean and variance. However, in reliability and degradation monitoring, data are often autocorrelated, time-dependent, and non-stationary. This assumption is highly restrictive and limits the applicability of the proposed method. The authors should justify it by examining the effect of autocorrelation, performing a robustness analysis, or explicitly acknowledging it as a limitation.*

Response:
This is a critical point, and we agree that the original presentation did not adequately address the implications of this assumption. In the revised manuscript the modeling framework has been extended to a state-space formulation, where process parameters evolve over time allows the model to capture nonstationary behavior. To assess dependence structure, an autocorrelation analysis has been included in the case study (Figure 7), showing that dependence is weak for the dataset considered. We have added a robustness discussion addressing the impact of mild autocorrelation on monitoring performance, and the role of adaptive smoothing in mitigating dependence effects. Importantly, we now explicitly acknowledge that strong autocorrelation may require further model extensions (e.g., ARMA-type state evolution), and we identify this as a direction for future research. Thus, the revised manuscript both relaxes the strict i.i.d. assumption and clearly delineates its scope of applicability.

***Comment 5:*** *The Linex loss is essentially used in an ad hoc manner. The authors themselves acknowledge that for the student-t distribution, the exponential moment may not exist, and for σ^2, the integrals diverge. Moreover, the Linex loss has no role in error control or ARL: it only affects point estimation, with no theoretical analysis of how a impacts ARL, no guidance on choosing a, and no robustness check against model misspecification. As a result, the Linex loss appears mostly decorative rather than effective, and its removal would likely have minimal impact on the results. The authors should clarify and justify why the Linex loss is included at all.*

Response:
We appreciate this critical observation and have carefully reconsidered the role of the Linex loss function. In the revised manuscript the use of Linex loss is now explicitly motivated as a tool for modeling asymmetric estimation risk, which can arise in applications where underestimation and overestimation have different practical consequences. We clearly separate estimation layer where Linex loss influences posterior point estimates, and Monitoring layer where control limits and ARL properties are determined independently. Additional clarification has been added to emphasize that the linex loss does not directly control ARL behavior, and its influence on detection performance is indirect, via estimation bias. To avoid any perception of arbitrariness, we provide guidance on parameter selection, and include discussion on its sensitivity and limitations. Importantly, the revised framework (DR-BAEWMA) is fully functional under standard squared-error loss, and Linex loss is now presented as an optional extension rather than a core requirement.

This repositioning ensures that the use of Linex loss is transparent, justified, and non-essential to the validity of the proposed method.

***Comment 6:*** *A serious concern is the negative estimates of σ^2. The paper itself states that “if β_t/(α_t-1)<τ^2 occurs, the estimator becomes negative … enforce a floor” which constitutes a direct violation of Bayesian principles, making the resulting estimator no longer truly Bayesian. This represents a significant weak spot in the proposed methodology.*

Response:
We agree that this was a serious weakness in the original version, and we have treated it as such. The revised manuscript no longer relies on an ad hoc floor to patch negative variance estimates. Instead, we now introduce a distributionally robust Bayesian updating mechanism based on Wasserstein ambiguity sets. This revision changes the logic of the estimation problem in an important way. The posterior update is no longer forced to produce unstable variance estimates through an arbitrary truncation rule. The robustified variance inflation term is derived from DRO duality, which ensures positivity in a principled manner. As a result, the estimator remains compatible with the Bayesian structure of the model while protecting against misspecification and contamination. In the revised text, we now explain clearly that the positivity of the updated variance is not achieved by post-processing or clipping, but by a mathematically justified robust update. This removes the earlier inconsistency and addresses the reviewer’s concern directly.

**Comment 7**: *The data are not publicly accessible; “upon reasonable request” is not acceptable for PLOS ONE*

Response: We understand this concern and have revised the data availability statement to comply with PLOS ONE requirements.In the revised manuscript. The data access statement has been updated to identify a non-author institutional contact point, consistent with journal policy. The source of the data is now described more precisely.

***Comment 8****: The definition of FEWMA is not clearly specified*

Response: We appreciate this observation. The term FEWMA was not defined clearly enough in the original manuscript. In the revised version FEWMA is now defined explicitly at first use as Frequentist EWMA, the abbreviation is included in the nomenclature table. Its role in the comparison study is described more carefully so that readers can distinguish it from the proposed Bayesian and distributionally robust versions. This clarification improves consistency and avoids confusion for readers who may not be familiar with the shorthand.

***Comment 9:*** *These issues should be addressed consistently throughout the paper.Some subsection headings appear without numbering and are presented only in boldface, which disrupts the structural consistency of the manuscript. For example, “Notation and Assumptions”, “Bayes Estimators under SELF” and “Variance of the Dispersion Estimator” are not numbered like other sections. Please ensure that all sections and subsections are consistently numbered throughout the manuscript.*

Response:
We agree that inconsistent heading structure makes the manuscript harder to follow and gives an impression of incomplete editing. The manuscript has now been thoroughly reorganized so that all main sections and subsections are consistently numbered. Unnumbered bold headings have been removed or integrated into the formal section structure. The internal hierarchy of the manuscript is now uniform from beginning to end.

**Comment 10:** *Several phrases and subsection titles are repeated verbatim in the manuscript. For example, “Bayes Estimators under LLF: Bayes Estimators under LLF” appears consecutively in duplicate form. Such repetitions suggest insufficient proofreading and affect the overall presentation quality. A thorough and careful revision of the entire manuscript is strongly recommended to eliminate redundancies and improve clarity and consistency.*

Response:

This issue has been corrected carefully throughout the revised manuscript. In particular, repeated subsection titles have been removed, duplicate phrases and repeated heading fragments have been eliminated. The text has been checked for unnecessary repetition and streamlined where needed. These changes were made not only to improve style, but also to strengthen the logic of presentation. A manuscript of this type benefits from a clean progression of ideas, and we have revised the structure with that in mind.

**Comment 11:** *Many sentences, particularly in the Introduction and Methodology sections, are excessively long and contain multiple ideas in a single sentence, which reduces clarity. The authors are encouraged to split such sentences into shorter, more focused statements.*

**Response:**

We fully agree. This was one of the main readability issues in the earlier draft. The manuscript has now been edited to break long sentences into shorter, more direct statements, separate definitions from interpretation, and avoid packing several claims into a single sentence. This revision makes the argument easier to follow and reduces the risk of ambiguity, especially in the methodological sections where precision matters most.

**Comment 12:** *The manuscript frequently capitalizes common technical terms (e.g., Baseline Health State, Inspection Batch, Sensor Measurement Uncertainty) that are not proper nouns. Capitalization should be used consistently and limited to proper names or formally defined acronyms.*

Response:

We have standardized capitalization throughout the manuscript. Specifically, common technical expressions are no longer capitalized as if they were formal names. Proper nouns and officially defined acronyms are preserved, and terms such as baseline health state, inspection batch, and sensor measurement uncertainty are now used consistently in lower case unless introduced as formal definitions. This was a useful correction because inconsistent capitalization can make a paper look less disciplined than its underlying statistics actually are.

**Comment 13:** *Several statements use strong, promotional language (e.g., “offers a principled tool,” “ensures robustness”) without sufficient empirical or theoretical support. A more neutral, evidence-based scientific tone is recommended.*

Response:

We agree with this point and have revised the language accordingly. Phrases that sounded overly assertive or promotional have been replaced by more measured scientific wording. For example, claims of “principled” or “robust” behavior are now supported by specific results, rather than stated as general slogans, and interpretive statements are now tied more closely to the simulation and case study evidence. The revised manuscript now aims for a more restrained and evidence-based tone, which we believe better suits a statistical paper.

**Comment 14:** *Verb tense usage is inconsistent across sections, particularly between present and past tense in the Methods and Simulation sections. The authors should standardize tense usage.*

Response:

This has been corrected during the language revision. We now use tense more consistently present tense for general methodological statements and interpretation, past tense for simulation and case study findings, and present perfect or present tense where the paper describes what has been done in the revision. This improves clarity and makes the manuscript sound more coherent.

***Comment 15:*** *Several symbols and notations are introduced without immediate definition or are redefined later in the manuscript. All symbols should be clearly defined at their first occurrence and used consistently thereafter.*

Response:

We agree and have corrected this throughout the manuscript. Now, each symbol is defined at its first appearance, notation is collected in a dedicated nomenclature table, and repeated symbols are used consistently across sections. This is especially important in a paper like this, where the statistical development depends on a sequence of estimators, latent states, and robustified quantities. The revised version is much cleaner in this respect.

***Comment 16****: Some closely related concepts are described using different terms across sections (e.g., variability, dispersion, and volatility), which may confuse readers. The authors are encouraged to standardize terminology throughout the manuscript.*

Response:

We have standardized terminology throughout the paper. In particular:terms such as variability, dispersion, and volatility are now used with clear distinctions, the same concept is not described by different labels in different sections, and the revised manuscript uses a more stable statistical vocabulary. This helps prevent conceptual drift, which can otherwise confuse readers even when the mathematics is correct.

***Comment 17****: The manuscript contains scattered minor grammatical and typographical errors (e.g., missing articles, inconsistent hyphenation, spacing issues). A careful language edit is recommended to improve overall polish.*

Response:
We have performed a careful language edit across the manuscript to remove typographical and grammatical issues. This includes spacing errors, missing articles, punctuation inconsistencies, hyphenation problems, and minor syntax issues. The revised text is noticeably smoother and more polished as a result.

***Comment 18****: Figure and table captions should provide concise and clear titles that identify the content of the figure or table, rather than descriptive explanations. All figure and table captions should be carefully reviewed and revised accordingly.*

*Some equations in the manuscript are numbered while others are not. All mathematical expressions presented as displayed equations within the text should be consistently numbered***.**

Response: We agree and have revised all captions accordingly. In the new version, figure captions state clearly what the figure shows, table titles are concise and informative, and unnecessary descriptive wording has been removed from captions. This makes the visual material easier to scan and aligns better with journal style.

***Comment 19****: Some figure references in the manuscript are incorrect. For example, the text states “is compared in Figure 10,” whereas the correct reference should be Figure 11. Please carefully check and correct all in-text references throughout the manuscript.*

Response: This has been corrected. The revised manuscript now uses a consistent equation numbering scheme, all displayed mathematical expressions that are referred to in the text are numbered, numbering is sequential and complete, the text references the equations in a uniform way. This is especially important for the derivations in the methodology sections, where equation tracing must be straightforward.

***Comment 20:*** *The references should be presented in a uniform and correct format.*

*It is recommended to provide a table listing all abbreviations and their corresponding full terms. Throughout the manuscript, consistently use the abbreviations instead of repeatedly writing long expressions.*

Response:
We have checked all in-text figure references carefully and corrected them. In the revised manuscript, the figures are now cited in the correct order, forward references have been removed, and the numbering is aligned with the actual placement of the figures. This was a necessary correction because figure misreferences can seriously disrupt the reading flow and make the manuscript seem less carefully prepared than it is.

***Comment 21****: References should be presented in a uniform and correct format*

Response:
We have revised the reference list to ensure consistency in formatting. The updated reference section now follows a uniform style in terms of author presentation, journal title formatting, year and volume layout, DOI placement, and punctuation and spacing. We also checked the references cited in the revised sections to make sure they are fully integrated into the text and not simply appended.

*Comment 22: Provide a table listing all abbreviations and full terms*

Response:
We have added a dedicated nomenclature table listing all abbreviations and symbols used in the manuscript. This table now includes the main acronyms used in the methodological development, the simulation and case study abbreviations, and the key statistical symbols introduced in the derivations. This addition improves accessibility and makes the manuscript easier to navigate, especially for readers who may not be specialists in sequential Bayesian monitoring.

We are grateful for the reviewers’ insightful comments, which have led to a substantially improved manuscript in terms of rigor, clarity, and practical relevance. The introduction of the DR-BAEWMA framework, along with the expanded simulations, strengthened case study, and improved presentation, directly addresses the concerns raised during the review process.

We hope that the revisions meet the expectations of the Editor and the reviewers, and we would be pleased to provide any further clarification if needed.

Thank you again for your time and consideration.

Dr Bilal Himmat (CA)

On the behalf of all coauthors
