# Supplementary material for: A distributionally-robust bayesian adaptive EWMA chart for joint surveillance of lognormal process location and scale
Source: PLoS One. 2026 Jul 9;21(7):e0343029. doi: 10.1371/journal.pone.0343029 (PMC13349310; doi:10.1371/journal.pone.0343029)
Supplement: Peer review history

# Peer review: Decision Letter 0

**DOI:** 10.1371/journal.pone.0343029.r001

**Authors:** Arne Johannssen

20 Feb 2026

PONE-D-26-04764

A Bayesian Max-EWMA Chart for Joint Surveillance of Lognormal Process Location and Scale

PLOS One

Dear Dr. Himmat,

Thank you for submitting your manuscript to PLOS ONE. After careful consideration, we feel that it has merit but does not fully meet PLOS ONE’s publication criteria as it currently stands. Therefore, we invite you to submit a revised version of the manuscript that addresses the points raised during the review process.

Please submit your revised manuscript by Apr 06 2026 11:59PM. If you will need more time than this to complete your revisions, please reply to this message or contact the journal office at plosone@plos.org. When you're ready to submit your revision, log on to https://www.editorialmanager.com/pone/ and select the 'Submissions Needing Revision' folder to locate your manuscript file.

Please include the following items when submitting your revised manuscript:

- A letter that responds to each point raised by the academic editor and reviewer(s). You should upload this letter as a separate file labeled 'Response to Reviewers'.
- A marked-up copy of your manuscript that highlights changes made to the original version. You should upload this as a separate file labeled 'Revised Manuscript with Track Changes'.
- An unmarked version of your revised paper without tracked changes. You should upload this as a separate file labeled 'Manuscript'.

If you would like to make changes to your financial disclosure, please include your updated statement in your cover letter. Guidelines for resubmitting your figure files are available below the reviewer comments at the end of this letter.

If applicable, we recommend that you deposit your laboratory protocols in protocols.io to enhance the reproducibility of your results. Protocols.io assigns your protocol its own identifier (DOI) so that it can be cited independently in the future. For instructions see: https://journals.plos.org/plosone/s/submission-guidelines#loc-laboratory-protocols. Additionally, PLOS ONE offers an option for publishing peer-reviewed Lab Protocol articles, which describe protocols hosted on protocols.io. Read more information on sharing protocols at https://plos.org/protocols?utm_medium=editorial-email&utm_source=authorletters&utm_campaign=protocols.

We look forward to receiving your revised manuscript.

Kind regards,

Arne Johannssen

Academic Editor

PLOS One

**Journal Requirements:**

When submitting your revision, we need you to address these additional requirements.

1. Please ensure that your manuscript meets PLOS ONE's style requirements, including those for file naming. The PLOS ONE style templates can be found at

https://journals.plos.org/plosone/s/file?id=wjVg/PLOSOne_formatting_sample_main_body.pdf and

https://journals.plos.org/plosone/s/file?id=ba62/PLOSOne_formatting_sample_title_authors_affiliations.pdf

2. Please note that PLOS One has specific guidelines on code sharing for submissions in which author-generated code underpins the findings in the manuscript. In these cases, we expect all author-generated code to be made available without restrictions upon publication of the work. Please review our guidelines at https://journals.plos.org/plosone/s/materials-and-software-sharing#loc-sharing-code and ensure that your code is shared in a way that follows best practice and facilitates reproducibility and reuse.

3. In the online submission form you indicate that your data is not available for proprietary reasons and have provided a contact point for accessing this data. Please note that your current contact point is a co-author on this manuscript. According to our Data Policy, the contact point must not be an author on the manuscript and must be an institutional contact, ideally not an individual. Please revise your data statement to a non-author institutional point of contact, such as a data access or ethics committee, and send this to us via return email. Please also include contact information for the third party organization, and please include the full citation of where the data can be found.

4. We note you have included a table to which you do not refer in the text of your manuscript. Please ensure that you refer to Table 6 in your text; if accepted, production will need this reference to link the reader to the Table.

5. If the reviewer comments include a recommendation to cite specific previously published works, please review and evaluate these publications to determine whether they are relevant and should be cited. There is no requirement to cite these works unless the editor has indicated otherwise.

**Additional Editor Comments:**

Please carefully consider the suggestions raised by the three reviewers.

[Note: HTML markup is below. Please do not edit.]

Reviewers' comments:

Reviewer's Responses to Questions

**Comments to the Author**

1. Is the manuscript technically sound, and do the data support the conclusions?

The manuscript must describe a technically sound piece of scientific research with data that supports the conclusions. Experiments must have been conducted rigorously, with appropriate controls, replication, and sample sizes. The conclusions must be drawn appropriately based on the data presented.

Reviewer #1: Yes

Reviewer #2: Yes

Reviewer #3: Partly

**********

2. Has the statistical analysis been performed appropriately and rigorously?

Reviewer #1: Yes

Reviewer #2: Yes

Reviewer #3: N/A

**********

3. Have the authors made all data underlying the findings in their manuscript fully available?

The PLOS Data policy requires authors to make all data underlying the findings described in their manuscript fully available without restriction, with rare exception (please refer to the Data Availability Statement in the manuscript PDF file). The data should be provided as part of the manuscript or its supporting information, or deposited to a public repository. For example, in addition to summary statistics, the data points behind means, medians and variance measures should be available. If there are restrictions on publicly sharing data—e.g. participant privacy or use of data from a third party—those must be specified.

Reviewer #1: Yes

Reviewer #2: Yes

Reviewer #3: No

**********

4. Is the manuscript presented in an intelligible fashion and written in standard English?

PLOS ONE does not copyedit accepted manuscripts, so the language in submitted articles must be clear, correct, and unambiguous. Any typographical or grammatical errors should be corrected at revision, so please note any specific errors here.

Reviewer #1: Yes

Reviewer #2: Yes

Reviewer #3: Yes

**********

5. Review Comments to the Author

Please use the space provided to explain your answers to the questions above. You may also include additional comments for the author, including concerns about dual publication, research ethics, or publication ethics. (Please upload your review as an attachment if it exceeds 20,000 characters)

Reviewer #1: Revised version:

The paper presents a novel and interesting idea by applying the MAX-EWMA method to the lognormal distribution. I find the concept valuable and acceptable. However, a few minor points should be addressed.

Minor:

To further strengthen the context and breadth of your contribution, I recommend engaging with recent advancements in related fields. Specifically, the work would benefit from a discussion with studies focusing on risk-informed decision-making for critical infrastructure maintenance, advanced metaheuristic optimization techniques applied to real-world scheduling challenges, and the latest trends in AI-driven non-destructive evaluation and quality control in manufacturing using deep learning for defect detection. Additionally, incorporating insights from recent data-driven process modeling in metallurgy and materials science could provide valuable perspectives on generalizing your current findings or contrasting your methodology with established industrial prediction approaches. You can cite the following works:

• Zhou, N., Luo, L., Sheng, G., & Jiang, X. (2025). Scheduling the Imperfect Maintenance and Replacement of Power Substation Equipment: A Risk-Based Optimization Model. IEEE Transactions on Power Delivery, 40(4), 2154-2166. doi: 10.1109/TPWRD.2025.3572076

• Long, X., Cai, W., Yang, L., & Huang, H. (2024). Improved particle swarm optimization with reverse learning and neighbor adjustment for space surveillance network task scheduling. Swarm and Evolutionary Computation, 85, 101482. doi: https://doi.org/10.1016/j.swevo.2024.101482

• Xu, H., Han, F., Zhou, W., Liu, Y., Ding, F.,... Zhu, J. (2024). ESMNet: An enhanced YOLOv7-based approach to detect surface defects in precision metal workpieces. Measurement, 235, 114970. doi:https://doi.org/10.1016/j.measurement.2024.114970

• Xia, Y., Song, Q., Yi, B., Lyu, T., Sun, Z.,... Li, Y. (2025). Improving out-of-distribution generalization for online weld expulsion inspection using physics-informed neural networks. Welding in the World, 69(5),1309-1322. doi: 10.1007/s40194-025-01950-6

• Wang, G., Yang, Y., Zhou, S., Li, B., Wei, Y.,... Wang, H. (2024). Data Analysis and Prediction Model for Copper Matte Smelting Process. Metallurgical and Materials Transactions B, 55(4), 2552-2567. doi:10.1007/s11663-024-03115-0

I suggest to change the fonts of the figures including xticks and yticks and labels to time new roman.

A literature review table can be added.

Major:

The simulation section is well designed and implemented; no major revisions are required. My only suggestion is to highlight (e.g., bold) the best-performing method in each comparison table to enhance readability and understanding.

The case study section, however, requires further development. The dataset should be made available, and a step-by-step procedure should be provided for reproducibility. For example, a Kolmogorov–Smirnov (K–S) test can be used to demonstrate that the data follow a lognormal distribution. Additionally, including a flowchart to illustrate the practical problem-solving process would strengthen this section. Some exploratory data analysis (EDA)—such as a time-series plot for the illustrative example—would also improve clarity and insight.

Finally, the R or Python code used for simulations and data generation should be included in the supplementary material, along with sufficient explanations and comments to help readers understand and replicate the results.

Reviewer #2: Revised Reviewer Comments:

The paper presents an interesting idea; however, several clarifications and improvements are required.

1. Comparison with existing work:

The following paper also proposes a MAX-EWMA approach:

“Joint monitoring of mean and variance using Max-EWMA for Weibull process” by Muhammad Noor-ul-Amin, Irfan Aslam, and Navid Feroze.

Please clarify how your work differs from this study. In that paper, two statistics are defined, whereas in your paper such distinction is not clearly presented. Elaborate on this difference to better position your contribution.

2. Choice of distribution:

The paper employs the lognormal distribution. However, the Weibull model is frequently used in reliability surveillance studies, while others sometimes adopt the lognormal model. Please justify your selection of the lognormal distribution and consider conducting a goodness-of-fit test—such as the Kolmogorov–Smirnov (K–S) test—to validate this choice.

3. Simulation type:

Your study currently reports zero-state ARL results. It is also necessary to include a steady-state simulation to provide a more comprehensive performance assessment. Please add this analysis to the paper.

4. Model robustness:

The robustness analysis presented in Table 7 should also be evaluated under a Weibull distribution to verify the model’s stability and general applicability.

5. More discussions:

I think the authors can provide some other directions for this work as it can be applied in other fields. Based on my knowledge, I suggest to provide the following topics and cite the suggested works:

- Application of MAXXEWMA in quantile based moder: Hao, R., & Yang, X. (2024). Multiple-output quantile regression neural network. Statistics and Computing,34(2), 89.doi: 10.1007/s11222-024-10408-6 and Li, L., Xia, Y., Ren, S., & Yang, X. (2025). Homogeneity Pursuit in the Functional-Coefficient Quantile Regression Model for Panel Data with Censored Data, 29(3), 323-348. doi:10.1515/snde-2023-0024

- Fault detection with the proposed method: Wang, H., Li, Y., Men, T., & Li, L. (2024). Physically Interpretable Wavelet-Guided Networks With DynamicFrequency Decomposition for Machine Intelligence Fault Prediction. IEEE Transactions on Systems, Man and Cybernetics: Systems, 54(8), 4863-4875. doi: 10.1109/TSMC.2024.3389068 and Wan, A., Zhang, F., Al-Bukhaiti, K., Cheng, X., Ji, X., Wang, J.,... Shan, T. (2025). A Novel GA-PSO-SVM Model for Compound Fault Diagnosis in Gearboxes With Limited Data. IEEE Sensors Journal, 25(16),30443-30431.doi: 10.1109/JSEN.2025.3576761 and Lu, Y., Wang, S., Zhang, C., Chen, R., Dui, H., Mazurkiewicz, D.,... Zhang, Y. (2025). A dynamic imperfect inspection-based maintenance optimization considering dependent competing failure. Measurement, 253,117470. doi: https://doi.org/10.1016/j.measurement.2025.117470

Generally, I appreciate the authors and suggest the paper for publication after the revision round.

Reviewer #3: The paper proposes a Bayesian Max-EWMA framework for real-time monitoring of lognormal process mean and variance, accounting for measurement error and variable batch sizes, and demonstrates early detection of deviations and robustness in industrial applications. The topic is relevant and potentially valuable to the fields of statistical process control (SPC) and reliability monitoring.

However, in its current form, the manuscript suffers from significant scientific, methodological, validation, mathematical transparency, and presentation deficiencies. Several claims are made without formal justification or adequate comparative evaluation, and parts of the proposed methodology lack sufficient rigor or clear explanation.

Major comments:

The authors propose a Bayesian Max-EWMA framework for lognormal processes. However, Max-EWMA charts have been previously introduced, Bayesian EWMA charts are extensively studied, and the conjugate NIG model for normal mean–variance monitoring is standard. The manuscript should provide direct comparisons with state-of-the-art methods and include a table summarizing theoretical and practical contributions relative to existing studies. It should also clarify the role of key components, such as measurement-error correction, the Max statistic, and Linex loss integration.

The manuscript does not provide a clear and precise definition of the Baseline Health State model.

Please pay careful attention to the structure and writing of the manuscript. Several obvious writing errors are observed, and the paper requires a thorough revision.

Examples include:

Page 3, line 76: “1.3 Joint monitoring and Max-type statistics Joint monitoring” – it is unclear what “1.3” refers to.

Page 17, line 365: “the per-Inspection Batch estimators at time under the Baseline Health State model.” – there is no space between “ ” and “under”. Similar errors appear elsewhere in the manuscript.

Page 21, line 458: “The objective is to monitor for shifts in the underlying process parameters ( , 2)” – the notation for the parameters is incorrect.

These are just a few examples of writing issues. Careful attention is required to correct such errors throughout the manuscript.

The study assumes that the observations X_it are independent and identically distributed (i.i.d.) following a normal distribution with constant mean and variance. However, in reliability and degradation monitoring, data are often autocorrelated, time-dependent, and non-stationary. This assumption is highly restrictive and limits the applicability of the proposed method. The authors should justify it by examining the effect of autocorrelation, performing a robustness analysis, or explicitly acknowledging it as a limitation.

The Linex loss is essentially used in an ad hoc manner. The authors themselves acknowledge that for the student-t distribution, the exponential moment may not exist, and for σ^2, the integrals diverge. Moreover, the Linex loss has no role in error control or ARL: it only affects point estimation, with no theoretical analysis of how a impacts ARL, no guidance on choosing a, and no robustness check against model misspecification. As a result, the Linex loss appears mostly decorative rather than effective, and its removal would likely have minimal impact on the results. The authors should clarify and justify why the Linex loss is included at all.

A serious concern is the negative estimates of σ^2. The paper itself states that “if β_t/(α_t-1)<τ^2 occurs, the estimator becomes negative … enforce a floor” which constitutes a direct violation of Bayesian principles, making the resulting estimator no longer truly Bayesian. This represents a significant weak spot in the proposed methodology.

The data are not publicly accessible. PLOS ONE places strong emphasis on Data Availability, yet the authors state that the data are available only “upon reasonable request.” This is generally not acceptable for PLOS ONE and limits reproducibility.

The definition of FEWMA is not clearly specified.

These issues should be addressed consistently throughout the paper.

Some subsection headings appear without numbering and are presented only in boldface, which disrupts the structural consistency of the manuscript. For example, “Notation and Assumptions”, “Bayes Estimators under SELF” and “Variance of the Dispersion Estimator” are not numbered like other sections. Please ensure that all sections and subsections are consistently numbered throughout the manuscript.

Several phrases and subsection titles are repeated verbatim in the manuscript. For example, “Bayes Estimators under LLF: Bayes Estimators under LLF” appears consecutively in duplicate form. Such repetitions suggest insufficient proofreading and affect the overall presentation quality. A thorough and careful revision of the entire manuscript is strongly recommended to eliminate redundancies and improve clarity and consistency.

Many sentences, particularly in the Introduction and Methodology sections, are excessively long and contain multiple ideas in a single sentence, which reduces clarity. The authors are encouraged to split such sentences into shorter, more focused statements.

The manuscript frequently capitalizes common technical terms (e.g., Baseline Health State, Inspection Batch, Sensor Measurement Uncertainty) that are not proper nouns. Capitalization should be used consistently and limited to proper names or formally defined acronyms.

Several statements use strong, promotional language (e.g., “offers a principled tool,” “ensures robustness”) without sufficient empirical or theoretical support. A more neutral, evidence-based scientific tone is recommended.

Verb tense usage is inconsistent across sections, particularly between present and past tense in the Methods and Simulation sections. The authors should standardize tense usage.

Several symbols and notations are introduced without immediate definition or are redefined later in the manuscript. All symbols should be clearly defined at their first occurrence and used consistently thereafter.

Some closely related concepts are described using different terms across sections (e.g., variability, dispersion, and volatility), which may confuse readers. The authors are encouraged to standardize terminology throughout the manuscript.

The manuscript contains scattered minor grammatical and typographical errors (e.g., missing articles, inconsistent hyphenation, spacing issues). A careful language edit is recommended to improve overall polish.

Figure and table captions should provide concise and clear titles that identify the content of the figure or table, rather than descriptive explanations. All figure and table captions should be carefully reviewed and revised accordingly.

Some equations in the manuscript are numbered while others are not. All mathematical expressions presented as displayed equations within the text should be consistently numbered.

Some figure references in the manuscript are incorrect. For example, the text states “is compared in Figure 10,” whereas the correct reference should be Figure 11. Please carefully check and correct all in-text references throughout the manuscript.

The references should be presented in a uniform and correct format.

It is recommended to provide a table listing all abbreviations and their corresponding full terms. Throughout the manuscript, consistently use the abbreviations instead of repeatedly writing long expressions.

**********

6. PLOS authors have the option to publish the peer review history of their article (what does this mean?). If published, this will include your full peer review and any attached files.

If you choose “no”, your identity will remain anonymous but your review may still be made public.

**Do you want your identity to be public for this peer review?**  For information about this choice, including consent withdrawal, please see our Privacy Policy.

Reviewer #1: **Yes:** Ali Yeganeh

Reviewer #2: No

Reviewer #3: No

**********

[NOTE: If reviewer comments were submitted as an attachment file, they will be attached to this email and accessible via the submission site. Please log into your account, locate the manuscript record, and check for the action link "View Attachments". If this link does not appear, there are no attachment files.]

To ensure your figures meet our technical requirements, please review our figure guidelines: https://journals.plos.org/plosone/s/figures

You may also use PLOS’s free figure tool, NAAS, to help you prepare publication quality figures: https://journals.plos.org/plosone/s/figures#loc-tools-for-figure-preparation.

NAAS will assess whether your figures meet our technical requirements by comparing each figure against our figure specifications.

Attachment Submitted filename: review.docx

# Author comment: Author response to Decision Letter 1

**DOI:** 10.1371/journal.pone.0343029.r002

29 Apr 2026

Response to Reviewers

Manuscript ID: PONE-D-26-04764

Title (Revised): A Distributionally-Robust Bayesian Adaptive EWMA Chart for Joint Surveillance of Lognormal Process Location and Scale

We sincerely thank the Academic Editor and the reviewers for their careful evaluation of our manuscript and for the constructive comments that have helped us improve both the clarity and the technical depth of the work. The original submission, titled “A Bayesian Max-EWMA Chart for Joint Surveillance of Lognormal Process Location and Scale,” presented a Bayesian extension of the Max-EWMA framework. However, in light of the reviewers’ concerns, particularly regarding novelty, robustness, and methodological rigor, we have undertaken a substantial revision of the manuscript.

In the revised version, we introduce a Distributionally-Robust Bayesian Adaptive EWMA (DR-BAEWMA) framework. This is not a superficial modification, but a meaningful extension that incorporates distributional robustness, adaptive state-space modeling, and a more principled treatment of uncertainty. These changes address the key concerns raised by the reviewers and significantly strengthen the contribution of the paper.

We have revised the manuscript thoroughly and respond to each comment in detail below.

Editor Comment 1: Compliance with PLOS ONE style requirements

Response:

We have carefully revised the manuscript to ensure full compliance with PLOS ONE formatting and style guidelines. Specifically, All section headings and subheadings have been consistently numbered. Figure and table captions have been standardized to concise, descriptive titles. Notation and formatting inconsistencies have been corrected throughout the manuscript. File naming conventions and manuscript structure have been aligned with the journal’s requirements.

Editor Comment 2: Code availability and reproducibility

Response:

We fully agree with the importance of reproducibility. In the revised manuscript, a complete reproducibility workflow has been added in Sections 6 and 7. The manuscript now clearly describes the Data preprocessing (Phase-I and Phase-II separation), Model calibration via nested Monte Carlo simulation and online monitoring implementation. The R code used for simulations and case study analysis has been prepared and will be provided as Supplementary Material, with detailed comments to facilitate reuse and verification.

Editor Comment 3: Data availability statement revision

Response:

We have revised the Data Availability Statement to comply with PLOS ONE policy.

Editor Comment 4: Missing in-text reference to Table 6

Response:

Thank you for noting this. We have corrected this oversight; Table 6 is now explicitly referenced in the text (Sections 6 and 7). The discussion surrounding Table 6 has been expanded to clarify its role in signal-source attribution and interpretability.

Response to Reviewer #1

We thank Reviewer #1 for the positive evaluation of our work and for the constructive suggestions to further strengthen the manuscript. All comments have been carefully addressed, as detailed below.

Comment 1: To further strengthen the context and breadth of your contribution, I recommend engaging with recent advancements in related fields. Specifically, the work would benefit from a discussion with studies focusing on risk-informed decision-making for critical infrastructure maintenance, advanced metaheuristic optimization techniques applied to real-world scheduling challenges, and the latest trends in AI-driven non-destructive evaluation and quality control in manufacturing using deep learning for defect detection. Additionally, incorporating insights from recent data-driven process modeling in metallurgy and materials science could provide valuable perspectives on generalizing your current findings or contrasting your methodology with established industrial prediction approaches. You can cite the following works:

• Zhou, N., Luo, L., Sheng, G., & Jiang, X. (2025). Scheduling the Imperfect Maintenance and Replacement of Power Substation Equipment: A Risk-Based Optimization Model. IEEE Transactions on Power Delivery, 40(4), 2154-2166. doi: 10.1109/TPWRD.2025.3572076

• Long, X., Cai, W., Yang, L., & Huang, H. (2024). Improved particle swarm optimization with reverse learning and neighbor adjustment for space surveillance network task scheduling. Swarm and Evolutionary Computation, 85, 101482. doi: https://doi.org/10.1016/j.swevo.2024.101482

• Xu, H., Han, F., Zhou, W., Liu, Y., Ding, F.,... Zhu, J. (2024). ESMNet: An enhanced YOLOv7-based approach to detect surface defects in precision metal workpieces. Measurement, 235, 114970. doi:https://doi.org/10.1016/j.measurement.2024.114970

• Xia, Y., Song, Q., Yi, B., Lyu, T., Sun, Z.,... Li, Y. (2025). Improving out-of-distribution generalization for online weld expulsion inspection using physics-informed neural networks. Welding in the World, 69(5),1309-1322. doi: 10.1007/s40194-025-01950-6

• Wang, G., Yang, Y., Zhou, S., Li, B., Wei, Y.,... Wang, H. (2024). Data Analysis and Prediction Model for Copper Matte Smelting Process. Metallurgical and Materials Transactions B, 55(4), 2552-2567. doi:10.1007/s11663-024-03115-0

Response:

We appreciate this insightful suggestion. To strengthen the contextual positioning of our work, the introduction has been expanded to incorporate recent developments in risk-informed decision-making for infrastructure maintenance, metaheuristic optimization in scheduling, AI-driven defect detection and non-destructive evaluation and Data-driven modeling in metallurgy and materials science. The suggested references have been carefully reviewed and incorporated where relevant.

Comment 2: Improve figure formatting (fonts, labels, etc.)

Response:

We have revised all figures to improve clarity and consistency: Fonts (including axis labels and tick marks) have been standardized to Times New Roman, as recommended. Figure layouts have been refined for readability. All graphical elements now follow a uniform style consistent with journal expectations.

Comment 3: Add a literature review table

Response:

We agree that a structured comparison enhances clarity. A comprehensive literature review table (Table 1) has been added. This table contrasts: Existing monitoring frameworks (Max-EWMA, Bayesian EWMA, AI-based methods, etc.). Their assumptions, robustness properties, and limitations, and The specific gap addressed by the proposed DR-BAEWMA framework

This addition strengthens the positioning and clarity of contributions.

Comment 4: The simulation section is well designed and implemented; no major revisions are required. My only suggestion is to highlight (e.g., bold) the best-performing method in each comparison table to enhance readability and understanding.

Response:

Thank you for this practical suggestion. All simulation result tables have been revised to highlight the best-performing methods (e.g., lowest ARL values) using bold formatting. This significantly improves readability and interpretability of the results.

Comment 5: Case study requires further development (data availability, step-by-step procedure, K–S test, EDA, flowchart)

Response:

We have substantially strengthened the case study section (Sections 6 and 7) to address this comment: Data Validation (K–S Test): A formal Kolmogorov–Smirnov test is now included to justify the lognormal assumption. Results are reported alongside graphical diagnostics. Exploratory Data Analysis (EDA): A comprehensive diagnostic figure (Figure 7) has been added, including: Time-series plots, Histograms (raw and log-transformed), Q–Q plots, Autocorrelation analysis. These revisions significantly enhance the clarity, transparency, and reproducibility of the case study.

Comment 6: Provide R/Python code for simulations

Response:

We fully agree with this recommendation. The simulation and implementation code has been, organized and documented with explanatory comments. It will be provided as Supplementary Material, ensuring full reproducibility of simulation experiments, case study analysis, and control-limit calibration

Comment: The simulation section is well designed; highlight best-performing methods in tables

Response:

We appreciate the reviewer’s positive assessment of the simulation design. To improve interpretability and readability, all simulation tables (now presented in Tables 4 and 5) have been revised to explicitly highlight the best-performing methods using bold formatting. The performance comparison is now more transparent, allowing readers to quickly identify minimum ARL values for out-of-control scenarios, and stability of in-control performance. These changes enhance the accessibility and clarity of the simulation results without altering their underlying structure.

Comment: The case study requires further development (dataset availability, step-by-step procedure, K–S test, EDA, flowchart, reproducibility)

Response:

We fully agree with the reviewer that the case study should serve as a transparent and reproducible demonstration of the proposed methodology. Accordingly, Sections 6 and 7 have been substantially revised and expanded. The key improvements are summarized below:

(a) Dataset transparency and availability

The dataset description has been clarified, including structure of inspection batches, and measurement context and preprocessing steps. The Data Availability Statement has been revised in accordance with journal policy.

(b) Step-by-step implementation procedure

A structured workflow has been introduced to guide replication, covering phase-I data selection and baseline estimation, prior hyperparameter elicitation, calibration of control limits via nested simulation, online monitoring using DR-BAEWMA, and Interpretation of signals and adaptive decisions. This procedure is described explicitly in the text and visually summarized in Figure 9.

(c) Goodness-of-fit validation (K–S test)

A formal Kolmogorov–Smirnov (K–S) test has been incorporated to validate the lognormal modeling assumption. The results confirm that the log-transformed data are consistent with normality. The graphical comparison between empirical and fitted distributions is provided in Figure 8, supporting the formal test.

(d) Exploratory Data Analysis (EDA)

A comprehensive EDA has been added, including time-series visualization, histograms (raw and log-transformed data), Q–Q plot for normality assessment, and Autocorrelation function (ACF). These diagnostics are integrated into a single multi-panel figure (Figure 7) and discussed in Section 7.1. The EDA provides both model validation and insight into process dynamics, addressing the reviewer’s concern.

(e) Flowchart for practical implementation

A workflow diagram (Figure 9) has been added to illustrate the full implementation pipeline.

This includes data preprocessing, prior specification, robust calibration, and monitoring and decision-making. The flowchart enhances the practical usability of the proposed framework.

(f) Reproducibility (code and implementation)

The computational procedures used in simulation experiments, control-limit calibration, case study analysis have been clearly documented. The R/Python code is prepared and will be provided as Supplementary Material, including detailed comments, modular structure for reuse, and reproducible scripts for all reported results

Comment: Include sufficient explanations in code to help replication

Response:

We fully agree with the importance of clear and well-documented code. All scripts have been revised to include inline comments explaining each step, clear labeling of inputs, parameters, and outputs and Modular functions for simulation generation, posterior updating, and control chart computation. A brief usage guide is also included in the supplementary material to assist readers in reproducing the results. This ensures that the computational aspects of the study are fully transparent and accessible.

Response to Reviewer #2

We thank the reviewer for the thoughtful and technically grounded comments. They go directly to the positioning and depth of the contribution, and addressing them has helped us sharpen both the methodology and its presentation. Below, we respond point-by-point.

Comment 1: Comparison with existing work:

The following paper also proposes a MAX-EWMA approach:

“Joint monitoring of mean and variance using Max-EWMA for Weibull process” by Muhammad No2or-ul-Amin, Irfan Aslam, and Navid Feroze.

Please clarify how your work differs from this study. In that paper, two statistics are defined, whereas in your paper such distinction is not clearly presented. Elaborate on this difference to better position your contribution.

Response: This is an important point, and we agree that the distinction needed to be made much clearer. In the revised manuscript, we now explicitly position our work relative to the Weibull-based MAX-EWMA study mentioned by the reviewer. The key difference is not just the underlying distribution, but the modeling philosophy and inferential structure. The existing Weibull MAX-EWMA approach is built in a classical SPC framework, where monitoring statistics are constructed directly from sample estimates of mean and variance (or their Weibull equivalents), typically under fixed distributional assumptions. In contrast, our revised framework, now formulated as Distributionally-Robust Bayesian Adaptive EWMA (DR-BAEWMA), operates within a Bayesian state-space setting, where the process parameters are treated as latent evolving states, inference is carried out via posterior updating rather than plug-in estimators, and uncertainty is explicitly modeled and propagated over time. More importantly, the current revision introduces distributional robustness via Wasserstein ambiguity sets, which fundamentally changes the problem. Instead of assuming the model is correctly specified (e.g., Weibull or lognormal), we allow for controlled deviations from the nominal model. The monitoring statistic is therefore calibrated under worst-case distributions within an ambiguity set, rather than a single assumed distribution.

Regarding the reviewer’s observation about “two statistics” in the existing work: In our framework, the separation between location and scale is preserved through standardized latent components Z_μand Z_σ, which are then combined through a Max-type aggregation. This is now explicitly clarified in the revised Section 4, where both components and their roles are formally defined and interpreted. To make this distinction transparent, we have added a comparative discussion in the Introduction and Methodology sections, and included a literature comparison table (Table 1) highlighting differences in inferential paradigm (classical vs Bayesian), treatment of uncertainty, robustness to misspecification, and adaptability and dynamic updating.

Overall, while both approaches fall under the broad umbrella of MAX-type EWMA monitoring, they address different statistical problems—ours being explicitly designed for robust, adaptive monitoring under model uncertainty.

Comment 2: Choice of distribution:The paper employs the lognormal distribution. However, the Weibull model is frequently used in reliability surveillance studies, while others sometimes adopt the lognormal model. Please justify your selection of the lognormal distribution and consider conducting a goodness-of-fit test—such as the Kolmogorov–Smirnov (K–S) test—to validate this choice.

Response: We appreciate this comment and agree that the choice of distribution must be justified empirically rather than assumed. In the revised manuscript, we have strengthened this aspect in two ways. (1) The empirical justification via goodness-of-fit testing a formal Kolmogorov–Smirnov (K–S) test has been conducted on the log-transformed Phase-I data. The test results indicate no significant deviation from normality on the log scale, supporting the lognormal assumption for the original data. This result is now reported explicitly in Section 7.1, with graphical support provided in Figure 8. (2) We complement the formal test with a set of exploratory diagnostics (Figure 7), including histograms, Q–Q plots, time-series visualization, and autocorrelation analysis. These provide

Attachment Submitted filename: Point by point response to the reviewers comments.docx

# Peer review: Decision Letter 1

**DOI:** 10.1371/journal.pone.0343029.r003

**Authors:** Arne Johannssen

19 May 2026

A Distributionally-Robust Bayesian Adaptive EWMA Chart for Joint Surveillance of Lognormal Process Location and Scale

PONE-D-26-04764R1

Dear Dr. Himmat,

We’re pleased to inform you that your manuscript has been judged scientifically suitable for publication and will be formally accepted for publication once it meets all outstanding technical requirements.

Within one week, you’ll receive an e-mail detailing the required amendments. When these have been addressed, you’ll receive a formal acceptance letter and your manuscript will be scheduled for publication.

An invoice will be generated when your article is formally accepted. Please note, if your institution has a publishing partnership with PLOS and your article meets the relevant criteria, all or part of your publication costs will be covered. Please make sure your user information is up-to-date by logging into Editorial Manager at Editorial Manager® and clicking the ‘Update My Information' link at the top of the page. For questions related to billing, please contact billing support.

If your institution or institutions have a press office, please notify them about your upcoming paper to help maximize its impact. If they’ll be preparing press materials, please inform our press team as soon as possible -- no later than 48 hours after receiving the formal acceptance. Your manuscript will remain under strict press embargo until 2 pm Eastern Time on the date of publication. For more information, please contact onepress@plos.org.

Kind regards,

Arne Johannssen

Academic Editor

PLOS One

Additional Editor Comments (optional):

Reviewers' comments:

Reviewer's Responses to Questions

**Comments to the Author**

1. If the authors have adequately addressed your comments raised in a previous round of review and you feel that this manuscript is now acceptable for publication, you may indicate that here to bypass the “Comments to the Author” section, enter your conflict of interest statement in the “Confidential to Editor” section, and submit your "Accept" recommendation.

Reviewer #1: All comments have been addressed

Reviewer #3: All comments have been addressed

**********

2. Is the manuscript technically sound, and do the data support the conclusions?

The manuscript must describe a technically sound piece of scientific research with data that supports the conclusions. Experiments must have been conducted rigorously, with appropriate controls, replication, and sample sizes. The conclusions must be drawn appropriately based on the data presented.

Reviewer #1: Yes

Reviewer #3: Yes

**********

3. Has the statistical analysis been performed appropriately and rigorously?

Reviewer #1: Yes

Reviewer #3: Yes

**********

4. Have the authors made all data underlying the findings in their manuscript fully available?

The PLOS Data policy requires authors to make all data underlying the findings described in their manuscript fully available without restriction, with rare exception (please refer to the Data Availability Statement in the manuscript PDF file). The data should be provided as part of the manuscript or its supporting information, or deposited to a public repository. For example, in addition to summary statistics, the data points behind means, medians and variance measures should be available. If there are restrictions on publicly sharing data—e.g. participant privacy or use of data from a third party—those must be specified.

Reviewer #1: Yes

Reviewer #3: Yes

**********

5. Is the manuscript presented in an intelligible fashion and written in standard English?

PLOS ONE does not copyedit accepted manuscripts, so the language in submitted articles must be clear, correct, and unambiguous. Any typographical or grammatical errors should be corrected at revision, so please note any specific errors here.

Reviewer #1: Yes

Reviewer #3: Yes

**********

6. Review Comments to the Author

Please use the space provided to explain your answers to the questions above. You may also include additional comments for the author, including concerns about dual publication, research ethics, or publication ethics. (Please upload your review as an attachment if it exceeds 20,000 characters)

Reviewer #1: (No Response)

Reviewer #3: The revised manuscript demonstrates substantial improvement in clarity, methodological soundness, and overall presentation quality. The authors have carefully and comprehensively addressed both the major and minor reviewer comments. In its current form, the paper meets the scientific standards of the journal and I consider it suitable for acceptance.

**********

7. PLOS authors have the option to publish the peer review history of their article (what does this mean?). If published, this will include your full peer review and any attached files.

If you choose “no”, your identity will remain anonymous but your review may still be made public.

**Do you want your identity to be public for this peer review?**  For information about this choice, including consent withdrawal, please see our Privacy Policy.

Reviewer #1: **Yes:** Ali Yeganeh

Reviewer #3: **Yes:** Sara Abossedgh

**********

# Editor report: Acceptance letter

**DOI:** 10.1371/journal.pone.0343029.r004

**Authors:** Arne Johannssen

PONE-D-26-04764R1

PLOS One

Dear Dr. Himmat,

I'm pleased to inform you that your manuscript has been deemed suitable for publication in PLOS One. Congratulations! Your manuscript is now being handed over to our production team.

At this stage, our production department will prepare your paper for publication. This includes ensuring the following:

* All references, tables, and figures are properly cited

* All relevant supporting information is included in the manuscript submission,

* There are no issues that prevent the paper from being properly typeset

You will receive further instructions from the production team, including instructions on how to review your proof when it is ready. Please keep in mind that we are working through a large volume of accepted articles, so please give us a few days to review your paper and let you know the next and final steps.

Lastly, if your institution or institutions have a press office, please let them know about your upcoming paper now to help maximize its impact. If they'll be preparing press materials, please inform our press team within the next 48 hours. Your manuscript will remain under strict press embargo until 2 pm Eastern Time on the date of publication. For more information, please contact onepress@plos.org.

You will receive an invoice from PLOS for your publication fee after your manuscript has reached the completed accept phase. If you receive an email requesting payment before acceptance or for any other service, this may be a phishing scheme. Learn how to identify phishing emails and protect your accounts at https://explore.plos.org/phishing.

If we can help with anything else, please email us at customercare@plos.org.

Thank you for submitting your work to PLOS ONE and supporting open access.

Kind regards,

PLOS ONE Editorial Office Staff

on behalf of

Profesor Arne Johannssen

Academic Editor

PLOS One
